# Multimorbidity, disease clusters and risk of all-cause and cause-specific mortality: a population-based prospective cohort study

**DOI:** 10.1038/s41598-025-25285-w

**Published:** 2025-11-21

**Authors:** Thomas J. Littlejohns, Wenyu Liu, Catherine M. Calvin, Lei Clifton, Jennifer A. Collister, Elżbieta Kuźma, David J. Hunter

**Affiliations:** 1 Nuffield Department of Population Health, University of Oxford, Oxford, OX3 7LF UK; 2 Nuffield Department of Primary Care Health Sciences, University of Oxford, Oxford, OX2 6GG UK; 3 Alberinen Krankenhaus/Albertinen Haus gGmbH, Academic Teaching Hospital of the Faculty of Medicine of the University of Hamburg, 22457 Hamburg, Germany; 4 Department of Epidemiology, Harvard TH Chan School of Public Health, Boston, MA 02115 USA; 5 Big Data Institute, University of Oxford, Old Road Campus, Oxford, OX3 7LF UK

**Keywords:** Risk factors, Epidemiology

## Abstract

The number of people living with ≥ two health conditions, termed multimorbidity, is increasing. We investigated the impact of multimorbidity on all-cause and cause-specific mortality risk in 502,370 UK Biobank participants aged 40 to 70 years. Participants attended an assessment centre between 2006 and 2010 and self-reported medical conditions during a nurse-led verbal interview. The presence of ≥ 2 long-term conditions from a preselected list of 43 conditions was used to define multimorbidity. In a training sample (80% of participants with multimorbidity), disease clusters were identified in four groups: women aged (1) 40–59 or (2) 60–70, and men aged (3) 40–59 or (4) 60–70. Mortality was ascertained from linkage to death records. Multivariate Cox proportional-hazards regression models were used to assess the association between multimorbidity and mortality adjusted for age, sex, ethnicity, socioeconomic status and education. Over a 16-year follow-up period (median = 13 years) dose–response associations were observed between number of multimorbid conditions and risk of all-cause mortality (n = 44,399 deaths), and particularly strong dose-response associations with cause-specific deaths due to cardiovascular and respiratory conditions. For women, a mental health/cancer/pain-related conditions cluster at ages 40–59 (Hazard Ratio [HR] = 2.61, 95% Confidence Interval [CI] 2.33–2.93), and a respiratory and pain-related conditions cluster at ages 60–70 (HR = 2.03, 95% CI 1.0–2.17), were associated with the greatest risk of mortality. For men, clusters of cardiometabolic conditions at ages 40–59 (HR = 3.43, 95% CI 3.14–3.74) and 60–70 (HR = 2.24, 95% CI 2.13–2.35) were associated with greater mortality risk. These findings suggest that understanding the impact of multimorbidity, and especially clusters of disease, is important for tailoring healthcare approaches for mortality risk reduction.

**Supplementary Information:**

The online version contains supplementary material available at 10.1038/s41598-025-25285-w.

## Introduction

The number of people worldwide with two or more long-term health conditions, known as multimorbidity, is rising^1^. In the UK, the proportion of individuals aged 65 years or older with multimorbidity is estimated to increase from 54 to 68% by 2035^2^. In 2020, the UK Chief Medical Officers described multimorbidity as one of the greatest challenges facing the healthcare profession^3^. They emphasised that clinical training and practice needs to shift towards focusing on multiple, rather than individual, diseases and that a better understanding of the impact of disease clusters is necessary to improve patient outcomes^3^.

Multimorbidity is heterogeneous, and varies substantially between individuals. Understanding which diseases commonly cluster together and how these clusters affect mortality risk is essential for the identification of high-risk groups and effectively allocating healthcare resources^3–5^. Multimorbidity is associated with a greater risk of mortality, however, previous studies have typically only conceptualised multimorbidity either by its presence or absence, or by number of conditions, without further exploration of different clusters within multimorbidity presence^6^. Studies which have investigated clusters of multimorbid conditions have consisted of small sample sizes or focused on a narrow selection of health conditions with low prevalence^7–13^. A study of 113,000 primary care patient records found differential associations between clusters of multimorbidity and mortality at various ages, and there is a need to follow-up these findings in a population-based setting with detailed and standardised data collection^14^ Further, whilst multimorbidity has been associated with vascular and cancer mortality, these outcomes contain distinct subtypes, including diseases that are largely sex-specific, such as breast and prostate cancer^15^. Focusing on more granular causes of mortality will help identify whether there are differential associations between multimorbidity and pathologically distinct causes of mortality and provide insights into long-term prognosis.

In the current study, we addressed these knowledge gaps using a cohort of 500,000 participants with 44,000 deaths occurring over 16 years to investigate whether number of multimorbid conditions, and sex- and age- specific disease clusters, were associated with all-cause and cause-specific mortality.

## Methods

### Population

Subjects were participants in UK Biobank (UKB), a population-based cohort study which recruited half a million women and men aged 40–69 years from England, Scotland and Wales between 2006 and 2010^16^. At recruitment, all participants attended a baseline assessment centre where they provided sociodemographic, lifestyle and medical history information through a touchscreen questionnaire and nurse-led verbal interview, and underwent physical examinations. All participants provided electronic signed consent at the assessment centre. UK Biobank received ethical approval from the National Health Service North West Centre for Research Ethics Committee (Ref: 11/NW/0382).

### Multimorbidity

Participants self-reported health conditions during the baseline verbal interview, where the nurse asked participants what serious illnesses and disabilities they have been diagnosed by a doctor. To ensure standardised recording, nurses inputted answers electronically and were guided by a tree-based structure based on the *International Classification of Diseases, Tenth Revision* (*ICD-10*) coding system. Health conditions included in the multimorbidity definition were based on a list of diseases developed by Jani et al. to ascertain multimorbidity using the UK Biobank cohort (see Table S1 for list of conditions)^15^*.* Jani et al.’s selection of conditions was informed by Barnett et al.’s definition of multimorbidity which was developed to ascertain the distribution and prevalence of multimorbidity in a UK-based population in 2007^17^. This date aligns with when health conditions were assessed between 2006 and 2010 in UKB. Multimorbidity presence was defined as having two or more of the 43 included health conditions, with multimorbidity absence defined as having zero or one condition. The number of multimorbid conditions was derived with the following categories: ‘0–1’ , ‘2’, ‘3’, ‘4’, and ‘5+’. Disease clusters were identified in participants with multimorbidity using latent class analysis, described in detail in the ‘statistical analysis’ section. The reference group for all cluster analyses were participants without multimorbidity (0–1 health conditions).

### Mortality

Mortality was ascertained through linkage to electronic death registry records. NHS England provided death data for England and Wales, and the NHS Central Register; the National Records of Scotland provided death data for Scotland. The records include date of death from April 2006 to 30th November 2022, and the underlying causes of death classified using ICD-10 codes. Outcomes included all-cause mortality and cause-specific deaths based on categorisations used by the Office for National Statistics (ONS) to produce mortality statistic reports^18^ and grouping causes into ‘cancer mortality’ (ICD-10 chapter C), ‘vascular mortality’ (ICD-10 chapter I), and ‘other-cause mortality’ (non-cancer and non-vascular deaths).

### Covariates

UKB collected information on age and sex at recruitment. Townsend deprivation score was used as an indicator of socioeconomic status and was assigned to each participant corresponding to their residential postcode at recruitment^19^. Participants self-reported ethnicity, educational qualifications, smoking status, alcohol intake and physical activity through the touchscreen questionnaire. Standard alcohol units (alcohol by volume equivalents) were derived based on the number of typical volume drinks for each type of alcohol consumed per week. Physical activity was assessed using questions adapted for the touchscreen questionnaire from the validated short International Physical Activity Questionnaire^20^. Time spent in vigorous, moderate and walking activity was weighted by the energy expended for these categories of activity, to produce total metabolic equivalent task minutes per week. Body mass index (BMI) was derived from weight (kg) using scales and standing height (metres) measured during the physical examination.

### Statistical analysis

The distribution of baseline characteristics by number of multimorbid conditions were explored both unadjusted and standardised for age. Cox proportional-hazards regression models were used to assess the association between the number of multimorbidity conditions and all-cause mortality. Follow-up time in years was calculated from date of attending baseline assessment to whichever censoring date occurred first for death, loss-to follow-up, or end of follow-up. End of follow-up was defined as the last date of death registry data availability (30th November 2022). In the main model (Model A), analyses were adjusted for age, sex (Women, Men), ethnicity (White, Black, South Asian, Mixed, Other), Townsend deprivation score (in quintiles) and education (Primary, Secondary, Post-secondary non-tertiary, Tertiary). To account for lifestyle factors, the analyses were repeated (Model B) with additional adjustment for smoking status (Never, Former, Current), alcohol intake in units per week, BMI (Underweight, Normal, Overweight, Obese) and physical activity (Low, High). The proportional hazards assumption was visually assessed using scaled Schoenfeld residuals. There was no evidence that any of the variables included in the analyses violated the proportional hazards assumption. In a sensitivity analysis investigating the impact of short-term all-cause mortality, we re-ran the model excluding participants who died or were censored within the first two years of follow-up. The number of deaths per cause based on ONS categorisations were calculated, and the main analyses repeated for the association between number of multimorbid conditions and the top 10 causes of death. We repeated the main analysis for all-cause mortality stratified by sex (women, men) and age (40–59, 60–70), and plotted age-specific cumulative incidence of all-cause mortality by number of multimorbid conditions in women and men. We also repeated the analyses for cause-specific mortality accounting for the competing risk of mortality not caused by the outcome of interest using the Fine-Gray subdistribution hazard model.

Latent class analysis was used to determine disease clusters, allocating each participant with multimorbidity to a single non-overlapping cluster^14,21^. Clusters were estimated in women aged (1) 40–59 years, and (2) 60–70 years, and men aged (3) 40–59 years, and (4) 60–70 years. A randomly selected training data set of 80% of participants with multimorbidity was used to determine the optimal number of clusters within each group and to estimate the association of disease clusters with mortality. Statistics were generated for multiple latent class analysis models of 1 to 12 cluster solutions, and the optimal number of clusters were first determined using sample size–adjusted Bayesian Information Criteria statistics as well as capping the smallest cluster to greater than 5% of the training sample. We then used judgement and previous experience to finalise the selection of the clusters^21,22^. Each cluster within the four groups was assigned a label based on up to 3 health conditions with the highest probabilities of belonging to that cluster. Conditions were excluded from the labelling if the observed prevalence was equal to, or less than, that of the expected prevalence of the total population with multimorbidity; and/or their probabilities were less than 5% of contributing to the cluster. Cox proportional-hazards regression models were used to assess the association between each cluster and risk of all-cause, as well as cancer, vascular, and other-cause mortality. To assess the validity and generalisability of the determined cluster solutions, conditions from the remaining 20% of men and women with multimorbidity were entered into latent class analysis models, setting the number of clusters to match the optimal numbers from the training data set. The characterization and relative size of the clusters determined from the training and test data sets were compared, as were their associations with mortality risk in Cox proportional hazards models. Finally, mortality incidence rates (IRs) per 1,000 person-years were calculated for each cluster.

P values were 2-sided, and the type I error rate for statistical significance was set at α = 0.05. Due to multiple comparisons, a subsequent Bonferroni correction was applied within each sex- and age-specific subgroup for the multimorbidity clusters and mortality analyses. Analyses were performed using Stata SE version 17.0 (StataCorp). R package poLCA version 1.6.0.1 was used to derive disease clusters, R package riskRegression was used for competing risk analyses, and R package ggsurvfit version 1.0.0 was used to obtain the cumulative incidence plots.

## Results

The final sample included 502,370 participants, of whom 165,125 (33%) had multimorbidity. As the number of multimorbid conditions increased, participants were more likely to be older, women, be of white ethnicity, live in an area of greater socioeconomic deprivation, be former or current smokers, be obese and have low physical activity levels. Little difference in alcohol intake was observed (Table S2). Findings were similar for age-standardised baseline characteristics, except for ethnicity, for which an increasing number of multimorbid conditions were observed in participants of black and South Asian, but not white, ethnicity (Table S3). The contrasting findings between unadjusted and age-standardised ethnicity is likely due to non-white participants being younger on average compared to white participants.

**Fig. 1:**
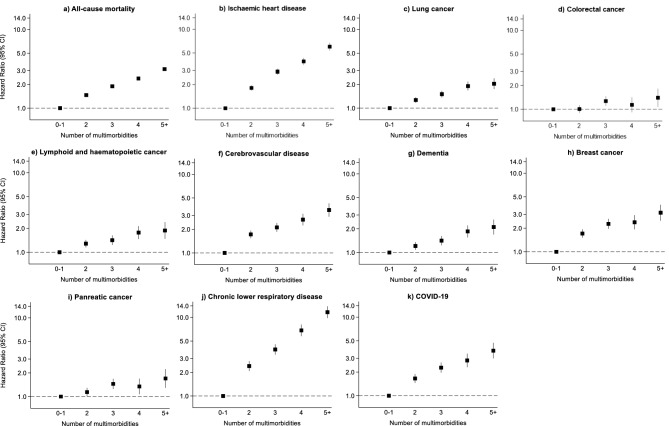
Association between number of multimorbid conditions with all-cause mortality and the top 10 primary causes of death in the UK Biobank population (adjusted for age, sex, ethnicity, Townsend deprivation index and education).

A total of 44,399 participants died of any cause over a median of 13 years (interquartile range = 13–14 years). The top ten causes of death were ischaemic heart disease (n = 4742), lung cancer (n = 3662), colorectal cancer (n = 2082), lymphoid and haematopoietic cancer (n = 1968), cerebrovascular disease (n = 1946), dementia (n = 1887), breast cancer (n = 1624), pancreatic cancer (n = 1612), chronic lower respiratory disease (n = 1457), and COVID-19 (n = 1416) (Table S4). For all-cause mortality, the Hazard Ratios (HR, 95% Confidence Intervals [CI]) were 1.47 (95% CI 1.43–1.50), 1.89 (95% CI 1.84–1.95), 2.38 (95% CI 2.30–2.47), and 3.14 (95% CI 3.01–3.27) for 2, 3, 4, and 5+ conditions, respectively, compared to 0–1 conditions (Fig. 1a). After excluding 2709 participants censored within 2 years of follow-up, including 2511 due to death, similar findings were observed with HRs of 1.45 (95% CI 1.42–1.49), 1.87 (95% CI 1.82–1.93), 2.32 (95% CI 2.24–2.41), and 3.10 (95% CI 3.00–3.24) for 2, 3, 4, and 5+ conditions, respectively, compared to 0–1 conditions. Dose–response associations were observed with the top 10 causes of death (Fig. 1b–k), with weaker associations observed with mortality caused by cancer, in particular colorectal and pancreatic cancer, and stronger associations observed with vascular and respiratory causes of mortality. The associations with all-cause and cause-specific mortality remained similar when adjusting for lifestyle factors (Table S5) and when accounting for the competing risk of causes of mortality not due to the outcome of interest (Table S6).

**Fig. 2:**
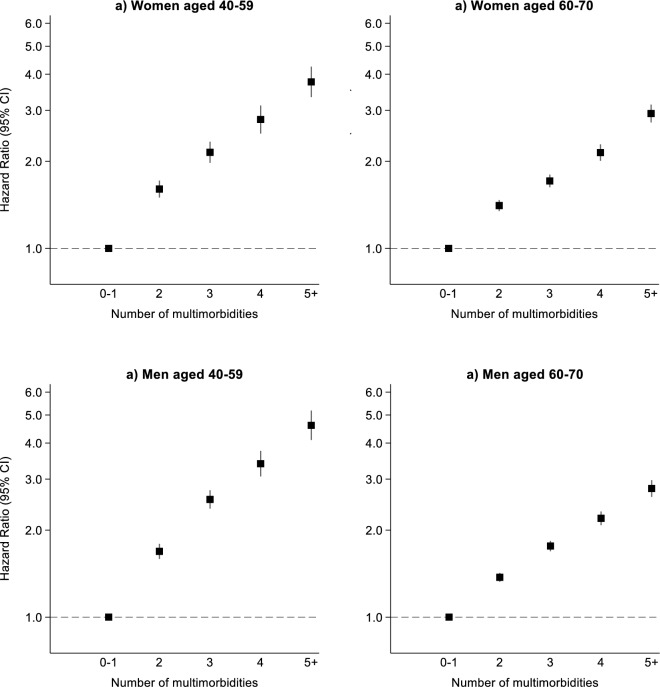
Association between number of multimorbid conditions with all-cause mortality by sex and age (adjusted for age, sex, ethnicity, Townsend deprivation index and education).

The age-specific cumulative incidence of all-cause mortality was greater for each increase in the number of multimorbid conditions, with the difference in risk emerging around 65 years of age in women, and 60 years in men (Fig. S1). Dose–response associations between number of multimorbid conditions and all-cause mortality were observed in women and men aged 40–59 and 60–70 (Fig. 2), and remained similar after adjustment for lifestyle factors (Table S7).

In the training sample, we identified six clusters as the optimal number for women aged 40–59 (n = 150,241) and 60–70 (n = 104,744) and men aged 40–59 (n = 120,382), and five clusters for men aged 60–70 (n = 93,968, Fig. S2). Clusters driven by cardiovascular and respiratory conditions were observed across all four groups (Tables S8–11). Clusters driven by mental health conditions were observed for both sexes aged 40–59, but not 60–70, whilst clusters driven by cancer were observed for both sexes aged 60–70, but not 40–59. Clusters driven by cardiometabolic conditions were observed for men of all ages, but not for women, whilst clusters driven by thyroid conditions were observed for women of all ages, but not for men.

All disease clusters were associated with an increased risk of all-cause mortality in women (Table 1) and men (Table 2). The strongest associations were observed in clusters driven by mental health, cancer and pain-related conditions (depression/cancer/dyspepsia; HR = 2.61, 95% CI 2.33–2.93) in women aged 40–59 and respiratory and pain-related conditions (asthma/pain/dyspepsia; HR = 2.03, 95% CI 1.90–2.17) in women aged 60–70. In men, the strongest associations were observed in clusters driven by cardiometabolic conditions for both 40–59 (diabetes/hypertension/CHD; HR = 3.43, 95% CI 3.14–3.74) and 60–70 (diabetes/hypertension/CHD; HR = 2.24, 95% CI 2.13–2.35) year olds. Associations remained similar with adjustment for lifestyle factors (Tables 1, 2). The majority of disease clusters were associated with a greater risk of cancer, vascular and other-cause mortality in women (Table S12) and men (Table S13). The associations for each cluster were generally stronger with vascular and other-cause of mortality, although as expected, clusters driven by cancer were more strongly associated with cancer mortality. There was a high degree of similarity in the characteristics of the cluster groups for men and women between the training and test samples (Tables S14 and S15).

**Table 1:** Association between multimorbidity clusters derived in training sample with all-cause mortality in women in UK Biobank.

Disease clusters	N	Deaths	Median morbidities (IQR)	All-cause mortality		Incidence rate per 1,000 person-years (95% CI)
				Model A^a^ HR (95% CI)	Model B^b^ HR (95% CI)	
40–59 years old at baseline^c^						
No multimorbidity	116,714	3017	0 (0–1)	1.00 (Reference)	1.00 (Reference)	1.9 (1.8–2.0)
Multimorbidity	33,527	1995	2 (2–3)	1.98 (1.87–2.10)	1.83 (1.72–1.94)	4.4 (4.2–4.6)
Hypertension (100%), diabetes (18%), CHD (7%)	11,296	811	2 (2–3)	2.20 (2.03–2.38)	2.02 (1.85–2.19)	5.3 (5.0–5.7)
Asthma (100%), psoriasis (17%), COPD (7%)	6247	292	2 (2–3)	1.73 (1.54–1.95)	1.59 (1.41–1.80)	3.4 (3.1–3.9)
Depression (38%), cancer (27%), dyspepsia (23%)	4534	329	2 (2–2)	2.61 (2.33–2.93)	2.38 (2.12–2.67)	5.4 (4.9–6.0)
Pain (100%), depression (25%), dyspepsia (23%)	4190	240	2 (2–3)	1.84 (1.62–2.11)	1.66 (1.45–1.89)	4.2 (3.7–4.8)
Thyroid conditions (100%), pain (22%), hypertension (21%)	3995	198	2 (2–3)	1.68 (1.45–1.94)	1.58 (1.36–1.82)	3.7 (3.2–4.2)
Migraine (100%), pain (30%), hypertension (20%)	3245	123	2 (2–3)	1.38 (1.15–1.65)	1.34 (1.12–1.61)	2.8 (2.3–3.3)
60–70 years old at baseline^c^						
No multimorbidity	65,007	5408	1 (0–1)	1.00 (Reference)	1.00 (Reference)	6.2 (6.0–6.3)
Multimorbidity	39,737	5792	2 (2–3)	1.66 (1.60–1.72)	1.59 (1.53–1.65)	11.1 (10.8–11.4)
Hypertension (100%), asthma (23%), diabetes (22%)	10,503	1804	2 (2–3)	1.91 (1.81–2.02)	1.82 (1.72–1.92)	13.2 (12.6–13.8)
Pain (61%), dyspepsia (35%), depression (21%)	6932	684	3 (2–4)	1.15 (1.06–1.25)	1.11 (1.03–1.21)	7.4 (6.9–8.0)
Cancer (100%), osteoporosis (8%)	6818	1149	2 (2–3)	2.00 (1.88–2.13)	1.95 (1.83–2.08)	13.2 (12.4–14.0)
Thyroid conditions (100%)	6133	761	3 (2–3)	1.41 (1.30–1.52)	1.34 (1.24–1.45)	9.4 (8.8–10.1)
Asthma (43%), pain (42%), dyspepsia (21%)	5647	998	2 (2–3)	2.03 (1.90–2.17)	1.90 (1.78–2.04)	13.6 (12.8–14.5)
Hypertension (100%), pain (100%)	3725	398	2 (2–3)	1.17 (1.05–1.29)	1.13 (1.02–1.25)^d^	8.0 (7.3–8.8)

CHD, coronary heart disease; CI, confidence interval; COPD, chronic obstructive pulmonary disease; HR, hazard ratio; IQR, interquartile range.

^a^Adjusted for age, sex, ethnicity, Townsend deprivation index and education.

^b^Adjusted for age, sex, ethnicity, Townsend deprivation index, education, body mass index, smoking, alcohol intake and physical activity.

^c^P-values Bonferroni corrected for the number of tests performed within each age-specific subgroup (age 40–59 and 60–70), with significance level set at 0.008 for 6 tests.

^d^Not statistically significant after applying Bonferroni correction.

**Table 2:** Association between multimorbidity clusters derived in training sample with all-cause mortality in men in UK Biobank.

Disease clusters	N	Deaths	Median morbidities (IQR)	All-cause mortality		Incidence rate per 1000 person-years (95% CI)
				Model A^a^ HR (95% CI)	Model B^b^ HR (95% CI)	
40–59 years old at baseline						
No multimorbidity	96,766	3889	0 (0–1)	1.00 (Reference)	1.00 (Reference)	2.9 (2.9–3.0)
Multimorbidity	23,616	2510	2 (2–3)	2.15 (2.05–2.27)	2.02 (1.92–2.13)	8.0 (7.7–8.3)
Hypertension (100%), pain (37%), CHD (17%)	7483	785	2 (2–3)	1.96 (1.81–2.11)	1.85 (1.71–2.00)	7.9 (7.3–8.4)
Asthma (100%), COPD (7%)	5185	437	2 (2–3)	1.88 (1.70–2.07)	1.81 (1.64–2.00)	6.3 (5.7–6.9)
Diabetes (100%), hypertension (83%), CHD (18%)	3574	631	3 (2–3)	3.43 (3.14–3.74)	3.17 (2.90–3.47)	13.8 (12.8–15.0)
Pain (53%), dyspepsia (36%), cancer (17%)	3500	345	2 (2–7)	1.99 (1.78–2.23)	1.89 (1.69–2.11)	7.5 (6.7–8.3)
Psoriasis (100%), asthma (45%), arthritis (6%)	2064	128	2 (2–3)	1.51 (1.27–1.80)	1.49 (1.25–1.77)	4.6 (3.9–5.4)
Depression (100%), pain (33%), dyspepsia (20%)	1801	180	3 (2–3)	2.07 (1.78–2.41)	1.85 (1.59–2.15)	7.5 (6.5–8.7)
60–70 years old at baseline						
No multimorbidity	58,749	8374	1 (0–1)	1.00 (Reference)	1.00 (Reference)	10.9 (10.7–11.1)
Multimorbidity	35,219	8544	2 (2–3)	1.63 (1.58–1.68)	1.56 (1.51–1.61)	19.2 (18.8–19.6)
Hypertension (100%), pain (38%), CHD (25%)	12,964	2783	2 (2–3)	1.40 (1.34–1.47)	1.36 (1.20–1.42)	16.9 (16.3–17.6)
Pain (51%), dyspepsia (32%), CHD (24%)	6307	1301	2 (2–3)	1.37 (1.29–1.45)	1.32 (1.25–1.40)	16.2 (15.4–17.1)
Diabetes (100%), hypertension (81%), CHD (28%)	6074	1960	3 (2–4)	2.24 (2.13–2.35)	2.04 (1.93–2.15)	26.8 (25.6–28.0)
Asthma (100%), COPD (13%), psoriasis (10%)	5520	1235	3 (2–3)	1.52 (1.43–1.61)	1.50 (1.41–1.60)	17.7 (16.7–18.7)
Cancer (100%)	4365	1265	2 (2–3)	2.01 (1.89–2.13)	1.96 (1.85–2.09)	24.2 (22.9–25.6)

CHD, coronary heart disease; CI, confidence interval; COPD, chronic obstructive pulmonary disease; HR, hazard ratio; IQR, interquartile range.

^a^Adjusted for age, sex, ethnicity, Townsend deprivation index and education.

^b^Adjusted for age, sex, ethnicity, Townsend deprivation index, education, body mass index, smoking, alcohol intake and physical activity.

^c^P-values Bonferroni corrected for the number of tests performed within each age-specific subgroup, with significance level set at 0.008 for 6 tests (age 40–59) and 0.01 for 5 tests (age 60–70).

## Discussion

### Main finding of this study

In this population-based cohort of half a million women and men aged 40–70 years, an increasing number of multimorbid conditions was associated with a greater risk of all-cause mortality, with the strongest associations observed for cardiovascular and respiratory causes of death. Different clusters of disease were identified in sex- and age-specific subgroups. For women, a mental health, cancer and pain-related cluster at ages 40–59, and a respiratory and pain-related conditions cluster at ages 60–70, were associated with greater risk of mortality, whilst for men, clusters of cardiometabolic conditions at all ages were associated with greater mortality risk. All associations were only slightly attenuated when accounting for lifestyle risk factors.

### What is already known on this topic and what this study adds

Our finding of a dose–response association between number of multimorbid conditions and all-cause mortality is consistent with previous population-based cohort studies conducted in a diversity of settings, including the UK^15,23^, United States^24^, China^25^, Chile^7^ and Iran^26^. Studies on cause-specific deaths, have found that multimorbidity is strongly associated with a greater risk of cardiovascular^15,25,27,28^, respiratory^25,28^ and ‘other’ causes of death^25,27^, and weakly associated with deaths due to cancer^15,25,27,28^. However, these definitions group together different causes of death with distinct aetiologies and pathological profiles. When using more granular definitions based on ONS categorisations for monitoring UK mortality rates, we found differential associations for cause-specific deaths. Stronger associations were observed for ischemic heart disease than for cerebrovascular disease, for chronic lower respiratory disease than for COVID-19, and for breast cancer than for pancreatic and colorectal cancer. Understanding the pathways underlying these risk differences may be important for the design of effective preventative, interventional and management approaches in individuals with multimorbidity.

It is important to consider the interplay between multimorbidity and demographic characteristics, as multimorbidity prevalence increases with age and is generally higher in women^29^. We found similar associations between number of multimorbid conditions and mortality in women and men, but stronger associations for younger (40–59 years) than older (60–70) ages. These findings of greater relative risk at younger ages are consistent with previous studies, with one hypothesis being that early onset disease is more aggressive^15,30^. However, the cumulative incidence (or absolute risk) of mortality is greater by multimorbidity status at older ages, which suggests that the attenuated relative risks in this age group are due to a higher background risk of mortality in those without multimorbidity. The distinction between relative and absolute risk is important, because, if causal, the impact of multimorbidity on mortality risk is greater at an individual-level in younger age groups, and at the population-level in older age groups.

Defining multimorbidity as number of conditions can provide insights into the overall burden of living with multiple health conditions. However, understanding which diseases commonly cluster together and the impact of these clusters on future health outcomes is essential for effective clinical management and resource allocation^3–5^. Two studies derived multimorbidity patterns in the China Kadoorie Biobank, a cohort of half a million Chinese women and men aged 30–79^25,31^. Both found that cardiometabolic and respiratory disease clusters were strongly associated with mortality. Although our results are similar, we found that the cardiometabolic cluster was strongly associated with mortality risk in men, whilst the respiratory cluster was strongly associated with mortality risk in older women. We also found evidence that a cluster characterised by mental health, cancer and pain-related conditions was associated with mortality in younger women. Disease clusters have been shown to vary substantially by sex and age^32,33^. From a clinical perspective, it is unsurprising that those with cancer or vascular disease at baseline are at high risk of cancer or vascular mortality, respectively. However, these participants were also at higher risk of mortality from other diseases, reinforcing the need to consider risk factor reduction and disease treatment beyond the specific disease that may represent the most obvious risk of death.

The mechanisms driving these associations are complex and multi-factorial^34^. Lifestyle factors likely play a key role on the causal pathways, both by increasing multimorbidity risk as well as mediating associations between multimorbidity and mortality. We adjusted for BMI, smoking, alcohol intake and physical activity, hypothesising that if these factors confound or mediate the associations then this will substantially attenuate the observed relationships^35^. However, all associations were only slightly weaker, including for clusters and cause-specific deaths strongly related to these factors, such as vascular and respiratory outcomes. Two studies, both in UKB, found that participants with multimorbidity and either following a healthy lifestyle^36^ or with high levels of physical activity^37^, had a lower mortality risk compared to those following an unhealthy lifestyle or low levels of physical activity, respectively. Longitudinal studies investigating the interplay between multimorbidity and lifestyle factors throughout the life-course are necessary to identify causal pathways and critical risk periods to inform targeted interventions.

### Strengths and limitations

Strengths of the study include a combination of a large sample size, cohort-wide linkage to death records, and detailed data collection. These factors enabled us to generate evidence on the association between multimorbidity with cause-specific mortality, investigate the role of sex- and age-specific disease clusters, and, account for various lifestyle factors. However, there are several limitations. First, UKB was not designed to be representative and participants are, on average, healthier than the general population^38^. Consequently, whilst clusters were validated using training and test sets, the identified clusters might not generalise to other populations. Second, self-report was used to ensure the ascertainment of health conditions was standardised, although this might have introduced underreporting or misclassification bias. A study based on a German population, found high agreement between 8 self-reported conditions and physician diagnoses, but found low agreement for arthritis^39^. In the UK-based English Longitudinal Study of Ageing (ELSA), half of respondents did not self-report a condition that was captured in their historical hospital records^40^. However, in ELSA, health conditions were reported in a questionnaire completed by participants, whereas in UKB, this information was obtained during a guided interview with trained nurses. Diagnoses obtained from primary care records could address certain limitations of self-reporting conditions, however, this data is currently only available for less than half of the UKB cohort. Third, despite the large sample size, there was a low prevalence of certain health conditions known to have substantial impacts on quality and disability-adjusted life years, including neurological diseases and mental health conditions. Fourth, multimorbidity varies based on the included conditions, and the Jani et al adaption of the Barnett definition might have missed important contributors to multimorbid clusters. Future studies should explore additional approaches for defining multimorbidity. Fifth, health conditions for the full sample were only self-reported at baseline assessment and we were unable to incorporate incident health conditions into the exposure definition. Understanding the trajectories of health conditions in populations with repeat measures could provide important insights into multimorbidity development and mortality risk. Sixth, residual confounding remains and causality cannot be determined due to the observational design of the current study. In the context of multimorbidity, in particular clusters which consist of a diverse range of conditions, the distinction between confounders and mediators warrants further exploration. For example, BMI might be on the causal pathway for multmorbid cardiometabolic conditions and a confounding factor for conditions less impacted by adiposity. Our findings were generally consistent when additionally adjusting for lifestyle factors, however, future research using longitudinal lifestyle measures could help elucidate the potential causal pathways underlying these associations.

## Conclusion

We found that an increasing number of multimorbid conditions was associated with a greater risk of all-cause mortality, with particularly strong dose–response associations observed with cardiovascular and respiratory causes of death. Our findings also highlight the importance of understanding the role of sex- and age-specific clusters of disease that affect various organ systems when assessing the impact of multimorbidity on future health outcomes.

## Supplementary Information

Supplementary Information.

## Data Availability

Data is from the UK Biobank (https://www.ukbiobank.ac.uk) under UKBB Resource Application ID 33952. All results presented in this manuscript, including the code used to generate them, will be returned to UK Biobank within 6 months of publication at which point they are made available for researchers to request (subject to UK Biobank approval). It is not feasible to share the data from us but from UKBB directly upon application approval. This is due to the UKBB restrictions that no individual-level data can be downloaded from the UK Biobank Research Analysis Platform (UKB-RAP) to a local machine. The cloud-based platform UKB-RAP is where a registered researcher can access the UKBB data and conduct their analyses.

## Code availability

The analyses were performed using STATA and R as stated in the Statistical analysis section. Codes are available upon request.
