# Supplementary material for: Multimorbidity, disease clusters and risk of all-cause and cause-specific mortality: a population-based prospective cohort study
Source: Sci Rep. 2025 Nov 21;15:41393. doi: 10.1038/s41598-025-25285-w (PMC12638925; doi:10.1038/s41598-025-25285-w)
Supplement: Supplementary file 1 — Supplementary Information. [file 41598_2025_25285_MOESM1_ESM.doc]

# Table S1. Prevalence of individual conditions by multimorbidity status

| **Condition** | **No multimorbidity**  **N (%)** | **Multimorbidity**  **N (%)** |
| --- | --- | --- |
| Hypertension | 43,546 (12.9) | 89,723 (54.3) |
| Painful condition | 24,501 (7.3) | 59,444 (36.0) |
| Asthma | 18,695 (5.5) | 39,563 (24.0) |
| Treated dyspepsia | 8,575 (2.5) | 30,484 (18.5) |
| Cancer (any) | 12,740 (3.8) | 25,868 (15.7) |
| Diabetes | 3,358 (1.0) | 22,134 (13.4) |
| Depression | 6,727 (2.0) | 21,727 (13.2) |
| Thyroid conditions | 7,878 (2.3) | 21,243 (12.9) |
| Coronary heart disease | 3,530 (1.1) | 19,190 (11.6) |
| Psoriasis or eczema | 4,960 (1.5) | 12,868 (7.8) |
| Migraine | 4,441 (1.3) | 9,939 (6.0) |
| Irritable bowel syndrome | 2,535 (0.8) | 8,952 (5.4) |
| Rheumatoid arthritis, other inflammatory polyarthropathies & systematic connective tissue disorders | 2,656 (0.8) | 8,370 (5.1) |
| Stroke and transient ischaemic attack | 1,267 (0.4) | 7,584 (4.6) |
| Chronic obstructive pulmonary disease | 1,099 (0.3) | 7,213 (4.4) |
| Anxiety & other neurotic, stress related & somatoform disorders | 1,880 (0.6) | 7,144 (4.3) |
| Osteoporosis | 1,778 (0.5) | 6,262 (3.8) |
| Prostate disorders (% in men) | 2,087 (1.3) | 6,169 (8.4) |
| Diverticular disease of intestine | 895 (0.3) | 4,506 (2.7) |
| Glaucoma | 1,339 (0.4) | 3,974 (2.4) |
| Endometriosis (% in women) | 1,163 (0.6) | 2,893 (3.2) |
| Atrial fibrillation | 714 (0.2) | 2,936 (1.8) |
| Inflammatory bowel disease | 1,358 (0.4) | 2,873 (1.7) |
| Epilepsy | 1,264 (0.4) | 2,787 (1.7) |
| Chronic sinusitis | 715 (0.2) | 2,387 (1.5) |
| Chronic fatigue syndrome | 523 (0.2) | 1,643 (1.0) |
| Schizophrenia (and related non-organic psychosis) or bipolar disorder | 535 (0.2) | 1,460 (0.9) |
| Pernicious anaemia | 234 (0.1) | 1,283 (0.8) |
| Chronic kidney disease | 99 (0.03) | 1,211 (0.7) |
| Multiple sclerosis | 693 (0.2) | 1,084 (0.7) |
| Meniere’s disease | 346 (0.1) | 1,030 (0.6) |
| Peripheral vascular disease | 236 (0.1) | 1,043 (0.6) |
| Bronchiectasis | 202 (0.1) | 933 (0.6) |
| Viral hepatitis | 486 (0.1) | 850 (0.5) |
| Parkinson's disease | 278 (0.1) | 579 (0.4) |
| Alcohol problems | 100 (0.03) | 708 (0.4) |
| Chronic liver disease | 247 (0.1) | 722 (0.4) |
| Heart failure | 121 (0.04) | 682 (0.4) |
| Polycystic ovary (% in women) | 171 (0.1) | 451 (0.5) |
| Treated constipation | 65 (0.02) | 338 (0.2) |
| Anorexia or bulimia | 82 (0.02) | 288 (0.2) |
| Dementia | 32 (0.01) | 92 (0.6) |
| Other psychoactive substance misuse | 18 (0.01) | 80 (0.1) |

**Table S2. Baseline characteristics by number of multimorbid conditions**

| **Characteristic** | **Total Population, N (%)**  **N=502,370** | **Number of multimorbid conditions, N (%)** | | | | |
| --- | --- | --- | --- | --- | --- | --- |
|  |  | **0-1**  **N=337,246** | **2**  **N=95,528** | **3**  **N=43,320** | **4**  **N=16,809** | **≥5**  **N=9,467** |
| **Age, mean (SD)** | 56.5 (8.1) | 55.3 (8.1) | 58.5 (7.6) | 59.7 (6.8) | 60.2 (7.0) | 60.4 (6.7) |
| **Women** | 273,301 (54.4) | 181,722 (53.9) | 51,971 (54.4) | 24,082 (55.6) | 9,693 (57.7) | 5,833 (61.6) |
| **Ethnicity** |  |  |  |  |  |  |
| White | 472,572 (94.6) | 316,448 (94.4) | 90,257 (94.9) | 40,957 (95.1) | 15,925 (95.2) | 8,985 (95.6) |
| Black | 8,058 (1.6) | 5,648 (1.7) | 1,470 (1.6) | 627 (1.5) | 209 (1.3) | 104 (1.1) |
| South Asian | 8,022 (1.6) | 5,290 (1.6) | 1,553 (1.6) | 723 (1.7) | 300 (1.8) | 156 (1.7) |
| Mixed | 2,953 (0.6) | 2,072 (0.6) | 534 (0.6) | 204 (0.5) | 104 (0.6) | 39 (0.4) |
| Other | 7,985 (1.6) | 5,812 (1.7) | 1,300 (1.4) | 571 (1.3) | 188 (1.1) | 114 (1.2) |
| **Townsend score** |  |  |  |  |  |  |
| 1 (least deprived) | 100,631 (20.1) | 70,695 (20.1) | 18,633 (19.5) | 7,494 (17.3) | 2,601 (15.5) | 1,208 (12.8) |
| 2 | 100,068 (19.9) | 68,982 (20.5) | 18,773 (19.7) | 8,089 (18.7) | 2,876 (17.1) | 1,348 (14.3) |
| 3 | 100,358 (20.0) | 68,415 (20.3) | 18,970 (19.9) | 8,302 (19.2) | 3,057 (18.2) | 1,614 (17.1) |
| 4 | 100,343 (20.0) | 66,934 (19.9) | 19,083 (20.0) | 8,854 (20.5) | 3,473 (20.7) | 1,999 (21.2) |
| 5 (most deprived) | 100,343 (20.0) | 61,801 (18.4) | 19,953 (20.9) | 10,524 (24.3) | 4,782 (28.5) | 3,283 (34.7) |
| **Education** |  |  |  |  |  |  |
| Primary | 86,022 (17.3) | 46,767 (14.0) | 19,925 (21.1) | 11,195 (26.2) | 4,967 (29.9) | 3,168 (34.0) |
| Secondary | 277,292 (55.8) | 195,817 (58.7) | 49,286 (52.3) | 20,612 (48.2) | 7,573 (45.7) | 4,004 (43.0) |
| Post-secondary non-tertiary | 44,302 (8.9) | 28,879 (8.7) | 8,880 (9.4) | 4,110 (9.6) | 1,560 (9.4) | 873 (9.4) |
| Tertiary | 89,230 (18.0) | 62,435 (18.7) | 16,221 (17.2) | 6,811 (15.9) | 2,489 (15.0) | 1,274 (13.7) |
| **Smoking status** |  |  |  |  |  |  |
| Never | 273,448 (54.8) | 192,950 (57.5) | 48,711 (51.3) | 20,513 (47.7) | 7,368 (44.1) | 3,906 (41.7) |
| Former | 173,009 (34.6) | 107,524 (32.1) | 36,378 (38.3) | 17,695 (41.1) | 7,292 (43.7) | 4,120 (43.4) |
| Current | 52,961 (10.6) | 34,837 (10.4) | 9,914 (10.4) | 4,834 (11.2) | 2,035 (12.2) | 1,341 (14.3) |
| **Units of alcohol intake, mean (SD)** | 11.5 (12.2) | 11.9 (12.1) | 11.2 (12.5) | 10.2 (12.4) | 9.1 (12.2) | 7.7 (11.7) |
| **BMI** |  |  |  |  |  |  |
| Underweight | 2,626 (0.5) | 1,874 (0.6) | 443 (0.5) | 180 (0.4) | 70 (0.4) | 59 (0.7) |
| Normal | 162,352 (32.5) | 124,039 (37.0) | 24,743 (26.0) | 9,078 (21.1) | 3,093 (18.6) | 1,399 (15.0) |
| Overweight | 212,062 (42.5) | 144,351 (43.1) | 40,824 (43.0) | 17,562 (40.8) | 6,171 (37.0) | 3,154 (33.7) |
| Obese | 122,222 (24.5) | 64,950 (19.4) | 28,998 (30.5) | 16,201 (37.7) | 7,328 (44.0) | 4,745 (50.7) |
| **High physical activity in METs** | 255,280 (63.5) | 179,383 (65.4) | 46,477 (61.9) | 19,374 (58.2) | 6,727 (53.5) | 3,319 (48.1) |

Abbreviations: BMI, Body Mass Index; METs, Metabolic Equivalent of Task; SD, Standard Deviation

# Table S3. Age-standardised baseline characteristics by number of multimorbid conditions

| **Characteristic** | **Number of multimorbid conditions, %** | | | | |
| --- | --- | --- | --- | --- | --- |
|  | **0-1**  **N=337,246** | **2**  **N=95,528** | **3**  **N=43,320** | **4**  **N=16,809** | **≥5**  **N=9,467** |
| **Female** | 53.7 | 55.1 | 56.9 | 60.0 | 63.7 |
| **Ethnicity** |  |  |  |  |  |
| White | 94.8 | 94.3 | 94.3 | 94.2 | 94.5 |
| Black | 1.5 | 1.8 | 1.7 | 1.6 | 1.4 |
| South Asian | 1.5 | 1.8 | 1.8 | 2.0 | 1.6 |
| Mixed | 0.6 | 0.6 | 0.6 | 0.8 | 0.6 |
| Other | 1.7 | 1.4 | 1.3 | 1.1 | 1.2 |
| **Townsend score** |  |  |  |  |  |
| 1 (least deprived) | 21.2 | 19.0 | 16.3 | 14.4 | 11.3 |
| 2 | 20.8 | 19.1 | 17.8 | 16.0 | 12.9 |
| 3 | 20.4 | 19.6 | 18.7 | 17.3 | 15.9 |
| 4 | 19.7 | 20.2 | 20.8 | 20.6 | 21.2 |
| 5 (most deprived) | 17.9 | 21.9 | 26.4 | 31.5 | 38.7 |
| **Education** |  |  |  |  |  |
| Primary | 15.4 | 18.7 | 22.3 | 25.5 | 29.4 |
| Secondary | 57.0 | 55.1 | 53.0 | 50.7 | 48.6 |
| Post-secondary non-tertiary | 9.0 | 8.9 | 8.9 | 8.5 | 8.2 |
| Tertiary | 18.7 | 17.2 | 15.6 | 15.3 | 13.8 |
| **Smoking status** |  |  |  |  |  |
| Never | 57.0 | 52.2 | 49.2 | 46.0 | 43.2 |
| Former | 33.0 | 36.5 | 37.8 | 39.3 | 39.3 |
| Current | 10.1 | 11.3 | 13.0 | 14.7 | 17.6 |
| **BMI** |  |  |  |  |  |
| Underweight | 5.5 | 4.9 | 4.6 | 4.9 | 8.6 |
| Normal | 36.7 | 26.5 | 21.5 | 19.2 | 15.5 |
| Overweight | 43.5 | 42.2 | 39.6 | 35.2 | 30.1 |
| Obese | 19.3 | 30.1 | 38.4 | 45.2 | 52.3 |
| **High physical activity in METs** | 65.7 | 61.3 | 57.4 | 52.5 | 47.2 |

Abbreviations: BMI, Body Mass Index; METs, Metabolic Equivalent of Task

# Table S4. Primary causes of death in UK Biobank based on the Office for National Statistics classifications

| **ICD-10 code** | **Underlying cause** | **N (% of deaths)** |
| --- | --- | --- |
| A00–A09 | Intestinal infectious diseases | 62 (0.1) |
| A15–A19, B90 | Tuberculosis | 7 (0.02 |
| A20, A44, A75–A79, A82–A84, A85.2, A90–A98, B50–B57 | Vector–borne diseases and rabies | 1 (0.0) |
| A33–A37, A49.2, A80, B01, B02, B05, B06, B15, B16, B17.0, B18.0, B18.1, B26, B91, G14 | Vaccine-preventable diseases | 8 (0.02) |
| A39, A87, G00–G03 | Meningitis and meningococcal infection | 15 (0.03) |
| A40–A41 | Septicaemia | 134 (0.3) |
| B20–B24 | Human immunodeficiency virus [HIV] disease | 19 (0.04) |
| C15 | Malignant neoplasm of oesophagus | 1,027 (2.3) |
| C16 | Malignant neoplasm of stomach | 518 (1.2) |
| C18–C21 | Malignant neoplasm of colon, sigmoid, rectum and anus | 2,082 (4.7) |
| C22 | Malignant neoplasm of liver and intrahepatic bile ducts | 736 (1.7) |
| C23-C24 | Malignant neoplasm of gallbladder and other parts of biliary tract | 163 (0.4) |
| C25 | Malignant neoplasm of pancreas | 1,612 (3.6) |
| C32 | Malignant neoplasm of larynx | 63 (0.1) |
| C33-C34 | Malignant neoplasm of trachea, bronchus and lung | 3,662 (8.2) |
| C40–C41 | Malignant neoplasms of bone and articular cartilage | 38 (0.1) |
| C43-C44 | Melanoma and other malignant neoplasms of skin | 470 (1.1) |
| C50 | Malignant neoplasm of breast | 1,624 (3.7) |
| C53–C55 | Malignant neoplasm of uterus | 378 (0.9) |
| C56 | Malignant neoplasm of ovary | 752 (1.7) |
| C61 | Malignant neoplasm of prostate | 1,284 (2.9) |
| C64 | Malignant neoplasm of kidney, except renal pelvis | 590 (1.3) |
| C67 | Malignant neoplasm of bladder | 509 (1.2) |
| C71 | Malignant neoplasm of brain | 940 (2.1) |
| C81–C96 | Malignant neoplasms, stated or presumed to be primary of lymphoid, haematopoietic and related tissue | 1,968 (4.4) |
| D00–D48 | In situ and benign neoplasms, and neoplasms of uncertain or unknown behaviour | 309 (0.7) |
| E10–E14 | Diabetes | 310 (0.7) |
| D50–D53, E40–E64 | Malnutrition, nutritional anaemias and other nutritional deficiencies | 3 (0.01) |
| E86–E87 | Disorders of fluid, electrolyte and acid–base balance (incl. dehydration) | 26 (0.06) |
| F01, F03, G30 | Dementia and Alzheimer disease | 1,887 (4.3) |
| F10–F19 | Mental and behavioural disorders due to psychoactive substance use | 41 (0.1) |
| G10–G12 | Systemic atrophies primarily affecting the central nervous system | 490 (1.1) |
| G20 | Parkinson disease | 571 (1.3) |
| G40-G41 | Epilepsy and status epilepticus | 47 (0.1) |
| G80–G83 | Cerebral palsy and other paralytic syndromes | 12 (0.03) |
| I05–I09 | Chronic rheumatic heart diseases | 80 (0.2) |
| I10–I15 | Hypertensive diseases | 322 (0.7) |
| I20–I25 | Ischaemic heart diseases | 4,742 (10.7) |
| I26–I28 | Pulmonary heart disease and diseases of pulmonary circulation | 251 (0.6) |
| I34–I38 | Nonrheumatic valve disorders and endocarditis | 292 (0.7) |
| I42 | Cardiomyopathy | 203 (0.5) |
| I46 | Cardiac arrest | 7 (0.02) |
| I47–I49 | Cardiac arrhythmias | 234 (0.5) |
| I50–I51 | Heart failure and complications and ill–defined heart disease | 297 (0.7) |
| I60–I69 | Cerebrovascular diseases | 1,946 (4.4) |
| I70 | Atherosclerosis | 6 (0.01) |
| I71 | Aortic aneurysm and dissection | 414 (0.9) |
| J00–J06, J20–J22 | Acute respiratory infections other than influenza and pneumonia | 86 (0.2) |
| J09–J18 | Influenza and pneumonia | 728 (1.6) |
| J40–J47 | Chronic lower respiratory diseases | 1,457 (3.3) |
| J80–J84 | Pulmonary oedema and other interstitial pulmonary diseases | 767 (1.7) |
| J96 | Respiratory failure | 1 (0.0) |
| K35–K46, K56 | Appendicitis, hernia and intestinal obstruction | 170 (0.4) |
| K70–K76 | Cirrhosis and other diseases of liver | 659 (1.5) |
| M00–M99 | Diseases of the musculoskeletal system and connective tissue | 251 (0.6) |
| N00–N39 | Diseases of the urinary system | 306 (0.7) |
| O00–O99 | Pregnancy, childbirth and the puerperium | 1 (0.0) |
| P00–P96 | Certain conditions originating in the perinatal period | 0 (0.0) |
| Q00–Q99 | Congenital malformations, deformations and chromosomal abnormalities | 73 (0.2) |
| V01–X59 | Accidents | 622 (1.4) |
| U07.1, U07.2 | COVID-19 | 1,416 (3.2) |

Abbreviations: ICD, International Classification of Diseases

# Table S5. Association between number of multimorbid conditions with all-cause mortality and top 10 primary causes of death in the UK Biobank population with additional adjustment for lifestyle factors

| **Cause of death** | **Number of multimorbid conditions, N (%)** | | | | | | | | | |
| --- | --- | --- | --- | --- | --- | --- | --- | --- | --- | --- |
|  | **Model A^a^ (HR 95% CI)** | | | | | **Model B^b^ (HR 95% CI)** | | | | |
|  | **0-1** | **2** | **3** | **4** | **5** | **0-1** | **2** | **3** | **4** | **5** |
| **All-cause mortality** | 1.00 (Reference) | 1.47 (1.43-1.50) | 1.89 (1.84-1.95) | 2.38 (2.30-2.47) | 3.14 (3.01-3.27) | 1.00 (Reference) | 1.43 (1.40-1.46) | 1.80 (1.75-1.85) | 2.22 (2.14-2.30) | 2.83 (2.71-2.96) |
| **Cause-specific mortality** |  |  |  |  |  |  |  |  |  |  |
| **Ischaemic heart**  **disease** | 1.00 (Reference) | 1.81  (1.68-1.95) | 2.91 (2.68-3.15) | 3.90 (3.52-4.33) | 6.01 (5.38-6.72) | 1.00 (Reference) | 1.69 (1.57-1.82) | 2.58 (2.38-2.80) | 3.33 (3.00-3.70) | 4.86 (4.34-5.44) |
| **Lung cancer** | 1.00 (Reference) | 1.27 (1.17-1.38) | 1.51 (1.36-1.66) | 1.91 (1.68-2.17) | 2.04 (1.75-2.39) | 1.00 (Reference) | 1.25 (1.15-1.36) | 1.44 (1.31-1.59) | 1.79 (1.58-2.04) | 1.85 (1.58-2.17) |
| **Colorectal cancer** | 1.00 (Reference) | 1.01 (0.90-1.12) | 1.27 (1.11-1.46) | 1.14 (0.92-1.42) | 1.40 (1.08-1.82) | 1.00 (Reference) | 0.98 (0.88-1.10) | 1.23 (1.07-1.41) | 1.09 (0.88-1.36) | 1.33 (1.02-1.74) |
| **Lymphoid and**  **haematopoietic cancer** | 1.00 (Reference) | 1.28 (1.15-1.43) | 1.42 (1.23-1.63) | 1.77 (1.47-2.13) | 1.88 (1.47-2.39) | 1.00 (Reference) | 1.26 (1.13-1.41) | 1.38 (1.20-1.59) | 1.70 (1.40-2.05) | 1.77 (1.39-2.26) |
| **Cerebrovascular**  **disease** | 1.00 (Reference) | 1.72 (1.54-1.92) | 2.11 (1.85-2.40) | 2.65 (2.24-3.15) | 3.51 (2.89-4.27) | 1.00 (Reference) | 1.73 (1.55-1.93) | 2.10 (1.84-2.39) | 2.60 (2.19-3.09) | 3.37 (2.76-4.11) |
| **Dementia** | 1.00 (Reference) | 1.21 (1.08-1.36) | 1.41 (1.24-1.62) | 1.85 (1.55-2.21) | 2.11 (1.69-2.62) | 1.00 (Reference) | 1.25 (1.12-1.40) | 1.47 (1.28-1.69) | 1.94 (1.62-2.32) | 2.20 (1.76-2.74) |
| **Breast cancer** | 1.00 (Reference) | 1.71 (1.51-1.93) | 2.27 (1.96-2.62) | 2.38 (1.93-2.93) | 3.14 (2.49-3.97) | 1.00 (Reference) | 1.66 (1.47-1.88) | 2.16 (1.87-2.51) | 2.22 (1.80-2.74) | 2.88 (2.27-3.65) |
| **Pancreatic cancer** | 1.00 (Reference) | 1.14 (1.01-1.29) | 1.45 (1.25-1.68) | 1.34 (1.07-1.69) | 1.70 (1.29-2.23) | 1.00 (Reference) | 1.11 (0.98-1.25) | 1.39 (1.19-1.61) | 1.27 (1.10-1.60) | 1.58 (1.20-2.08) |
| **Chronic lower**  **respiratory disease** | 1.00 (Reference) | 2.40 (2.08-2.78) | 3.90 (3.35-4.56) | 6.83 (5.76-8.10) | 11.64 (9.80-13.83) | 1.00 (Reference) | 2.49 (2.15-2.88) | 4.00 (3.42-4.67) | 6.82 (5.74-8.11) | 11.31 (9.49-13.49) |
| **COVID-19** | 1.00 (Reference) | 1.66 (1.45-1.89) | 2.28 (1.96-2.65) | 2.81 (2.31-3.43) | 3.75 (2.99-4.71) | 1.00 (Reference) | 1.51 (1.32-1.72) | 1.94 (1.67-2.26) | 2.27 (1.86-2.78) | 2.85 (2.26-3.59) |

Abbreviations: CI, Confidence Interval; HR, Hazard Ratio

^a^ Adjusted for age, sex, ethnicity, Townsend deprivation index and education

^b^ Adjusted for age, sex, ethnicity, Townsend deprivation index, education, body mass index, smoking, alcohol intake and physical activity

# Table S6. Association between number of multimorbid conditions with top 10 primary causes of death accounted for competing risk (using Fine and Gray’s subdistribution hazard model) in the UK Biobank population with additional adjustment for lifestyle factors

| **Cause of death** | **Number of multimorbid conditions, N (%)** | | | | |
| --- | --- | --- | --- | --- | --- |
|  | **Model A^a^ (HR 95% CI)** | | | | |
|  | **0-1** | **2** | **3** | **4** | **5** |
| **Ischaemic heart disease** | 1.00 (Reference) | 1.76 (1.63-1.91) | 2.82 (2.58-3.07) | 3.62 (3.25-4.04) | 5.44 (4.84-6.12) |
| **Lung cancer** | 1.00 (Reference) | 1.24 (1.14-1.35) | 1.44 (1.30-1.59) | 1.75 (1.53-2.00) | 1.78 (1.51-2.10) |
| **Colorectal cancer** | 1.00 (Reference) | 1.00 (0.89-1.12) | 1.26 (1.09-1.45) | 1.11 (0.88-1.38) | 1.26 (0.95-1.67) |
| **Lymphoid and haematopoietic cancer** | 1.00 (Reference) | 1.26 (1.12-1.41) | 1.36 (1.17-1.58) | 1.64 (1.35-2.01) | 1.85 (1.44-2.37) |
| **Cerebrovascular disease** | 1.00 (Reference) | 1.67 (1.49-1.88) | 2.07 (1.81-2.38) | 2.44 (2.03-2.92) | 3.19 (2.60-3.92) |
| **Dementia** | 1.00 (Reference) | 1.14 (1.01-1.28) | 1.26 (1.09-1.46) | 1.67 (1.39-2.00) | 1.83 (1.46-2.29) |
| **Breast cancer** | 1.00 (Reference) | 1.65 (1.45-1.88) | 2.22 (1.91-2.59) | 2.34 (1.89-2.90) | 3.12 (2.45-3.96) |
| **Pancreatic cancer** | 1.00 (Reference) | 1.15 (1.01-1.31) | 1.39 (1.18-1.62) | 1.24 (0.97-1.59) | 1.54 (1.15-2.07) |
| **Chronic lower respiratory disease** | 1.00 (Reference) | 2.29 (1.96-2.67) | 3.65 (3.10-4.31) | 6.50 (5.44-7.77) | 10.06 (8.37-2.10) |
| **COVID-19** | 1.00 (Reference) | 1.62 (1.41-1.87) | 2.26 (1.93-2.65) | 2.55 (2.06-3.15) | 3.20 (2.51-4.08) |

Abbreviations: CI, Confidence Interval; HR, Hazard Ratio

^a^ Adjusted for age, sex, ethnicity, Townsend deprivation index and education

**Figure S1. Age-specific cumulative incidence of all-cause mortality by number of multimorbid conditions in women and men from age 50^a^**

| **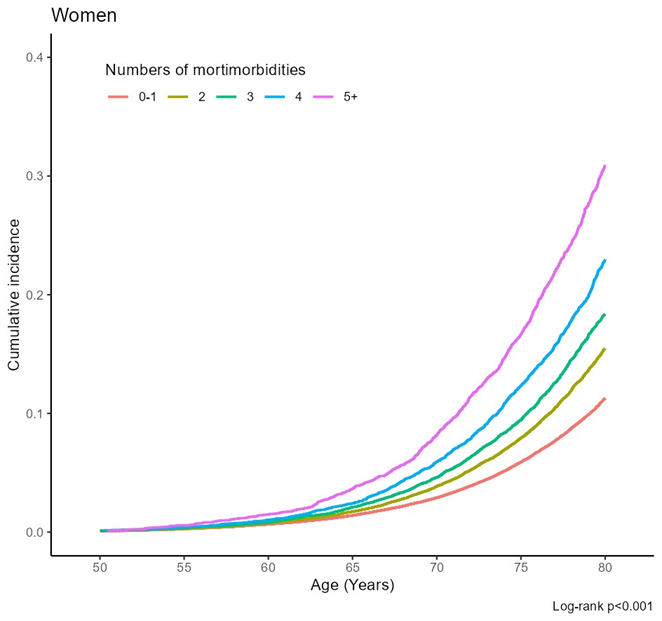** | **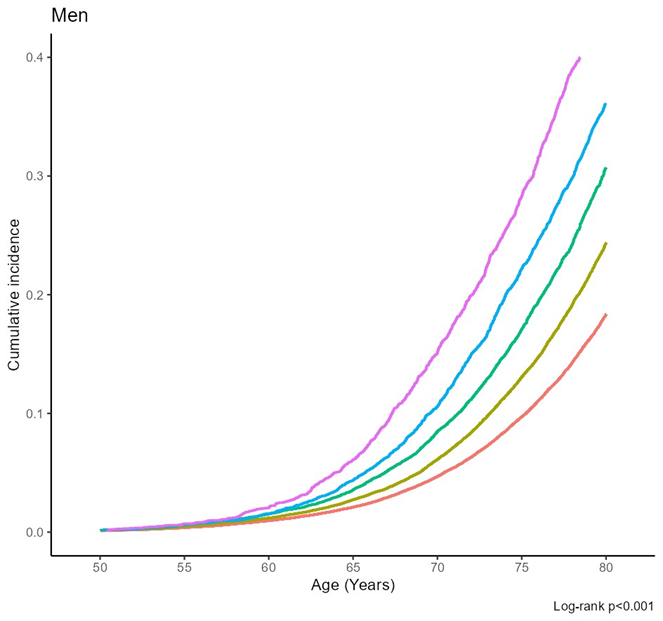** |
| --- | --- |

**Figure Legend:** ^a^ From age 50 due to the low number of deaths occurring between ages 40-49 years old

**Alt text:** A figure containing two plots, one for women and one for men, depicting the increase in all-cause mortality with age on the x-axis. Separate lines are used to show this increase within number of multimorbidities

# Table S7. Association between number of multimorbid conditions with all-cause mortality by sex and age with additional adjustment for lifestyle factors

| **Population** | **Number of multimorbid conditions, N (%)** | | | | | | | | | |
| --- | --- | --- | --- | --- | --- | --- | --- | --- | --- | --- |
|  | **Model A^a^ (HR 95% CI)** | | | | | **Model B^b^ (HR 95% CI)** | | | | |
|  | **0-1** | **2** | **3** | **4** | **5** | **0-1** | **2** | **3** | **4** | **5** |
| **Women aged 40-59** | 1.00 (Reference) | 1.60 (1.50-1.72) | 2.15 (1.98-2.34) | 2.79 (2.49-3.12) | 3.76 (3.33-4.25) | 1.00 (Reference) | 1.53 (1.43-1.64) | 1.98 (1.82-2.16) | 2.46 (2.20-2.76) | 3.20 (2.83-3.62) |
| **Women aged 60-70** | 1.00 (Reference) | 1.41 (1.35-1.47) | 1.71 (1.63-1.80) | 2.14 (2.01-2.29) | 2.92 (2.72-3.14) | 1.00 (Reference) | 1.38 (1.32-1.44) | 1.63 (1.55-1.72) | 2.01 (1.88-2.15) | 2.67 (2.48-2.87) |
| **Men aged 40-59** | 1.00 (Reference) | 1.69 (1.59-1.79) | 2.55 (2.37-2.75) | 3.39 (3.01-3.76) | 4.61 (4.10-5.18) | 1.00 (Reference) | 1.64 (1.54-1.74) | 2.34 (2.18-2.52) | 3.04 (2.75-3.37) | 3.97 (3.53-4.47) |
| **Men age 60-70** | 1.00 (Reference) | 1.37 (1.33-1.42) | 1.76 (1.69-1.84) | 2.20 (2.08-2.32) | 2.78 (2.61-2.98) | 1.00 (Reference) | 1.34 (1.30-1.39) | 1.69 (1.62-1.76) | 2.05 (1.94-2.17) | 2.54 (2.37-2.71) |

Abbreviations: CI, Confidence Interval; HR, Hazard Ratio

^a^ Adjusted for age, sex, ethnicity, Townsend deprivation index and education

^b^ Adjusted for age, sex, ethnicity, Townsend deprivation index, education, body mass index, smoking, alcohol intake and physical activity

# Figure S2. SABIC values for disease cluster solutions

| 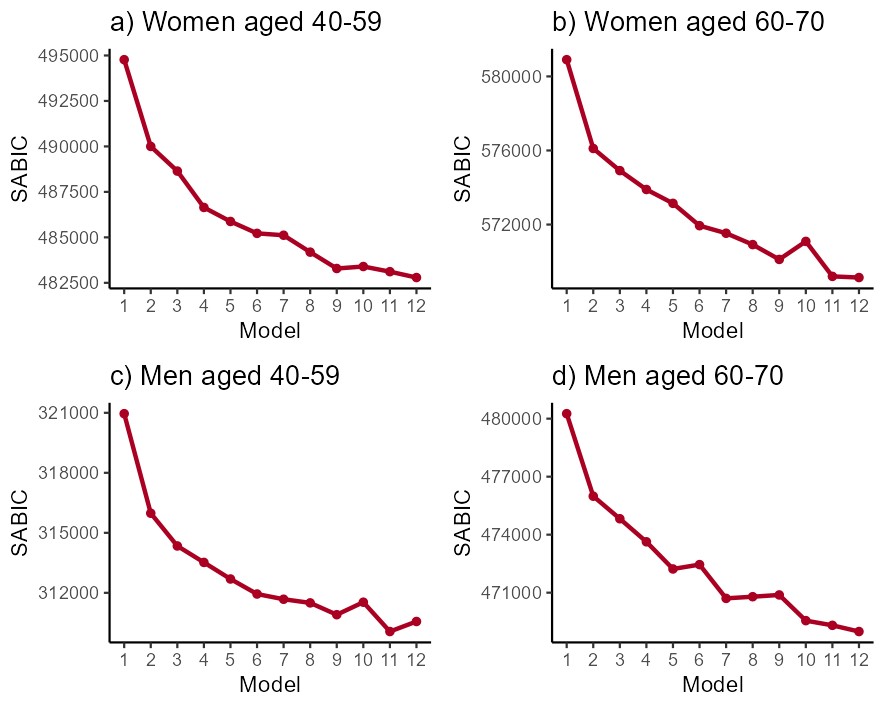  **Figure Legend:** Abbreviations: SABIC, Sample size – Adjusted Bayesian Information Criteria |  |
| --- | --- |
|  |  |
| **Alt Text:** A figure containing four plots by women and men and age groups used to select the appropriate number of multimorbidity clusters. Number of clusters are on the x-axis and the sample size – adjusted Bayesian information criteria is on the y-axis |  |
|  |  |

# Table S8. Probabilities and Observed vs Expected Ratios within 6 clusters derived in the training sample in women aged 40-59

| **Condition** |  | **Migraine, pain & hypertension** | | **Hypertension, diabetes & CHD** | | **Thyroid conditions, pain & hypertension** | | **Asthma, psoriasis & COPD** | | **Pain, depression & dyspepsia** | | **Depression, cancer & dyspepsia** | |
| --- | --- | --- | --- | --- | --- | --- | --- | --- | --- | --- | --- | --- | --- |
|  | **Expected** | **P** | **O/E** | **P** | **O/E** | **P** | **O/E** | **P** | **O/E** | **P** | **O/E** | **P** | **O/E** |
| Alcohol problems | 0.0029 | 0.0008 | 0.2759 | 0.0011 | 0.3793 | 0.0003 | 0.1034 | 0.0036 | 1.2414 | 0.0050 | 1.7241 | 0.0084 | 2.8966 |
| Anaemia | 0.0109 | 0.0053 | 0.4862 | 0.0074 | 0.6789 | 0.0237 | 2.1743 | 0.0084 | 0.7706 | 0.0081 | 0.7431 | 0.0151 | 1.3853 |
| Anorexia | 0.0053 | 0.0059 | 1.1132 | 0.0012 | 0.2264 | 0.0010 | 0.1887 | 0.0038 | 0.7170 | 0.0066 | 1.2453 | 0.0167 | 3.1509 |
| Anxiety | 0.0609 | 0.0580 | 0.9524 | 0.0305 | 0.5008 | 0.0390 | 0.6404 | 0.0448 | 0.7356 | 0.0718 | 1.1790 | 0.1427 | 2.3432 |
| Arthritis | 0.0616 | 0.0456 | 0.7403 | 0.0482 | 0.7825 | 0.0738 | 1.1981 | 0.0463 | 0.7516 | 0.0466 | 0.7565 | 0.1212 | 1.9675 |
| Asthma | 0.3069 | 0.1347 | 0.4389 | 0.2128 | 0.6934 | 0.1760 | 0.5735 | 1.0000 | 3.2584 | 0.0845 | 0.2753 | 0.0298 | 0.0971 |
| Atrial fibrillation | 0.0049 | 0.0030 | 0.6122 | 0.0062 | 1.2653 | 0.0054 | 1.1020 | 0.0024 | 0.4898 | 0.0046 | 0.9388 | 0.0076 | 1.5510 |
| Bronchiectasis | 0.0042 | 0.0009 | 0.2143 | 0.0016 | 0.3810 | 0.0018 | 0.4286 | 0.0121 | 2.8810 | 0.0019 | 0.4524 | 0.0059 | 1.4048 |
| Cancer | 0.1493 | 0.0965 | 0.6463 | 0.1240 | 0.8305 | 0.1380 | 0.9243 | 0.1225 | 0.8205 | 0.1377 | 0.9223 | 0.2714 | 1.8178 |
| CFS | 0.0185 | 0.0179 | 0.9676 | 0.0083 | 0.4486 | 0.0196 | 1.0595 | 0.0162 | 0.8757 | 0.0228 | 1.2324 | 0.0349 | 1.8865 |
| CHD | 0.0365 | 0.0048 | 0.1315 | 0.0703 | 1.9260 | 0.0200 | 0.5479 | 0.0190 | 0.5205 | 0.0250 | 0.6849 | 0.0314 | 0.8603 |
| Constipation | 0.0025 | 0.0021 | 0.8400 | 0.0013 | 0.5200 | 0.0006 | 0.2400 | 0.0014 | 0.5600 | 0.0050 | 2.0000 | 0.0043 | 1.7200 |
| COPD | 0.0326 | 0.0103 | 0.3160 | 0.0230 | 0.7055 | 0.0120 | 0.3681 | 0.0706 | 2.1656 | 0.0269 | 0.8252 | 0.0365 | 1.1196 |
| Dementia | 0.0003 | 0.0004 | 1.3333 | 0.0002 | 0.6667 | 0.0009 | 3.0000 | 0.0005 | 1.6667 | 0.0000 | 0.0000 | 0.0006 | 2.0000 |
| Depression | 0.2121 | 0.1900 | 0.8958 | 0.1429 | 0.6737 | 0.1719 | 0.8105 | 0.1747 | 0.8237 | 0.2526 | 1.1909 | 0.3820 | 1.8010 |
| Diabetes | 0.0805 | 0.0102 | 0.1267 | 0.1847 | 2.2944 | 0.0549 | 0.6820 | 0.0318 | 0.3950 | 0.0351 | 0.4360 | 0.0614 | 0.7627 |
| Drug abuse | 0.0004 | 0.0000 | 0.0000 | 0.0000 | 0.0000 | 0.0000 | 0.0000 | 0.0007 | 1.7500 | 0.0002 | 0.5000 | 0.0011 | 2.7500 |
| Dyspepsia | 0.1599 | 0.1372 | 0.8580 | 0.1322 | 0.8268 | 0.1182 | 0.7392 | 0.1364 | 0.8530 | 0.2153 | 1.3465 | 0.2328 | 1.4559 |
| Endometriosis | 0.0455 | 0.0545 | 1.1978 | 0.0260 | 0.5714 | 0.0316 | 0.6945 | 0.0420 | 0.9231 | 0.0534 | 1.1736 | 0.0821 | 1.8044 |
| Epilepsy | 0.0196 | 0.0173 | 0.8827 | 0.0142 | 0.7245 | 0.0135 | 0.6888 | 0.0167 | 0.8520 | 0.0189 | 0.9643 | 0.0411 | 2.0969 |
| Glaucoma | 0.0118 | 0.0055 | 0.4661 | 0.0122 | 1.0339 | 0.0089 | 0.7542 | 0.0074 | 0.6271 | 0.0126 | 1.0678 | 0.0183 | 1.5508 |
| Heart failure | 0.0021 | 0.0000 | 0.0000 | 0.0037 | 1.7619 | 0.0020 | 0.9524 | 0.0006 | 0.2857 | 0.0019 | 0.9048 | 0.0043 | 2.0476 |
| Hepatitis | 0.0050 | 0.0031 | 0.6200 | 0.0024 | 0.4800 | 0.0037 | 0.7400 | 0.0054 | 1.0800 | 0.0058 | 1.1600 | 0.0105 | 2.1000 |
| Hypertension | 0.3955 | 0.2041 | 0.5161 | 1.0000 | 2.5284 | 0.2092 | 0.5290 | 0.1620 | 0.4096 | 0.1456 | 0.3681 | 0.1164 | 0.2943 |
| IBD | 0.0192 | 0.0144 | 0.7500 | 0.0142 | 0.7396 | 0.0117 | 0.6094 | 0.0163 | 0.8490 | 0.0161 | 0.8385 | 0.0440 | 2.2917 |
| IBS | 0.0895 | 0.1090 | 1.2179 | 0.0421 | 0.4704 | 0.0508 | 0.5676 | 0.0779 | 0.8704 | 0.1293 | 1.4447 | 0.1672 | 1.8682 |
| Intestinal disease | 0.0197 | 0.0142 | 0.7208 | 0.0167 | 0.8477 | 0.0142 | 0.7208 | 0.0146 | 0.7411 | 0.0260 | 1.3198 | 0.0327 | 1.6599 |
| Kidney disease | 0.0070 | 0.0015 | 0.2143 | 0.0183 | 2.6143 | 0.0012 | 0.1714 | 0.0029 | 0.4143 | 0.0029 | 0.4143 | 0.0039 | 0.5571 |
| Liver disease | 0.0042 | 0.0017 | 0.4048 | 0.0020 | 0.4762 | 0.0041 | 0.9762 | 0.0021 | 0.5000 | 0.0048 | 1.1429 | 0.0104 | 2.4762 |
| Meniere’s disease | 0.0068 | 0.0078 | 1.1471 | 0.0046 | 0.6765 | 0.0040 | 0.5882 | 0.0042 | 0.6176 | 0.0097 | 1.4265 | 0.0117 | 1.7206 |
| Migraine | 0.1134 | 1.0000 | 8.8183 | 0.0234 | 0.2063 | 0.0303 | 0.2672 | 0.0577 | 0.5088 | 0.0359 | 0.3166 | 0.0059 | 0.0520 |
| Multiple sclerosis | 0.0115 | 0.0084 | 0.7304 | 0.0081 | 0.7043 | 0.0103 | 0.8957 | 0.0091 | 0.7913 | 0.0093 | 0.8087 | 0.0261 | 2.2696 |
| Osteoporosis | 0.0334 | 0.0200 | 0.5988 | 0.0185 | 0.5539 | 0.0329 | 0.9850 | 0.0271 | 0.8114 | 0.0388 | 1.1617 | 0.0682 | 2.0419 |
| Pain | 0.3385 | 0.2998 | 0.8857 | 0.2710 | 0.8006 | 0.2248 | 0.6641 | 0.2384 | 0.7043 | 1.0000 | 2.9542 | 0.0000 | 0.0000 |
| Parkinson’s disease | 0.0012 | 0.0002 | 0.1667 | 0.0007 | 0.5833 | 0.0008 | 0.6667 | 0.0011 | 0.9167 | 0.0007 | 0.5833 | 0.0026 | 2.1667 |
| Polycystic ovary | 0.0095 | 0.0077 | 0.8105 | 0.0065 | 0.6842 | 0.0098 | 1.0316 | 0.0093 | 0.9789 | 0.0084 | 0.8842 | 0.0162 | 1.7053 |
| Psoriasis | 0.0996 | 0.0925 | 0.9287 | 0.0458 | 0.4598 | 0.0510 | 0.5120 | 0.1711 | 1.7179 | 0.0979 | 0.9829 | 0.1587 | 1.5934 |
| PVD | 0.0071 | 0.0085 | 1.1972 | 0.0039 | 0.5493 | 0.0042 | 0.5915 | 0.0039 | 0.5493 | 0.0095 | 1.338 | 0.0156 | 2.1972 |
| Schizophrenia | 0.0112 | 0.0044 | 0.3929 | 0.0079 | 0.7054 | 0.0130 | 1.1607 | 0.0118 | 1.0536 | 0.0110 | 0.9821 | 0.0197 | 1.7589 |
| Sinusitis | 0.0195 | 0.0318 | 1.6308 | 0.0097 | 0.4974 | 0.0122 | 0.6256 | 0.0200 | 1.0256 | 0.0252 | 1.2923 | 0.0330 | 1.6923 |
| Stroke | 0.0270 | 0.0158 | 0.5852 | 0.0442 | 1.6370 | 0.0147 | 0.5444 | 0.0167 | 0.6185 | 0.0162 | 0.6000 | 0.0408 | 1.5111 |
| Thyroid conditions | 0.1826 | 0.0859 | 0.4704 | 0.1195 | 0.6544 | 1.0000 | 5.4765 | 0.0600 | 0.3286 | 0.0448 | 0.2453 | 0.0078 | 0.0427 |

Abbreviations: CFS, Chronic Fatigue Syndrome; CHD, Coronary Heart Disease; COPD, Chronic Obstructive Pulmonary Disease; IBD, Inflammatory Bowel Disease; IBS, Irritable Bowel Syndrome; O/E, Observed/Expected; P, Probability; PVD, Peripheral Vascular Disease

# Table S9. Probabilities and Observed vs Expected Ratios within 6 clusters derived in the training sample in women aged 60-70

| **Condition** |  | **Cancer & osteoporosis** | | **Hypertension, asthma & diabetes** | | **Thyroid conditions** | | **Asthma, pain, dyspepsia** | | **Hypertension & pain** | | **Pain, dyspepsia & depression** | |
| --- | --- | --- | --- | --- | --- | --- | --- | --- | --- | --- | --- | --- | --- |
|  | **Expected** | **P** | **O/E** | **P** | **O/E** | **P** | **O/E** | **P** | **O/E** | **P** | **O/E** | **P** | **O/E** |
| Alcohol problems | 0.0013 | 0.0012 | 0.9107 | 0.0005 | 0.3920 | 0.0000 | 0.0000 | 0.0012 | 0.9582 | 0.0000 | 0.0000 | 0.0035 | 2.7184 |
| Anaemia | 0.0103 | 0.0020 | 0.1974 | 0.0068 | 0.6565 | 0.0205 | 1.9881 | 0.0169 | 1.6432 | 0.0040 | 0.3887 | 0.0090 | 0.8734 |
| Anorexia | 0.0009 | 0.0007 | 0.7604 | 0.0005 | 0.5575 | 0.0003 | 0.3493 | 0.0014 | 1.5362 | 0.0000 | 0.0000 | 0.0021 | 2.3312 |
| Anxiety | 0.0390 | 0.0228 | 0.5835 | 0.0274 | 0.7034 | 0.0186 | 0.4777 | 0.0256 | 0.6567 | 0.0060 | 0.1527 | 0.0967 | 2.4792 |
| Arthritis | 0.0670 | 0.0339 | 0.5054 | 0.0681 | 1.0162 | 0.0614 | 0.9170 | 0.1442 | 2.1518 | 0.0180 | 0.2687 | 0.0459 | 0.6848 |
| Asthma | 0.2157 | 0.1297 | 0.6014 | 0.2292 | 1.0625 | 0.1286 | 0.5964 | 0.4307 | 1.9967 | 0.0744 | 0.3449 | 0.1687 | 0.7821 |
| Atrial fibrillation | 0.0155 | 0.0105 | 0.6783 | 0.0236 | 1.5245 | 0.0151 | 0.9762 | 0.0225 | 1.4538 | 0.0039 | 0.2534 | 0.0084 | 0.5414 |
| Bronchiectasis | 0.0091 | 0.0039 | 0.4323 | 0.0073 | 0.7997 | 0.0013 | 0.1382 | 0.033 | 3.6239 | 0.0011 | 0.1232 | 0.0019 | 0.2129 |
| Cancer | 0.2040 | 1.0000 | 4.9020 | 0.0874 | 0.4283 | 0.1049 | 0.5141 | 0.0957 | 0.469 | 0.0000 | 0.0000 | 0.0662 | 0.3246 |
| CFS | 0.0093 | 0.0050 | 0.5346 | 0.0057 | 0.6085 | 0.0072 | 0.7762 | 0.0076 | 0.8131 | 0.0000 | 0.0000 | 0.0213 | 2.2874 |
| CHD | 0.0877 | 0.0381 | 0.4343 | 0.1473 | 1.6794 | 0.0765 | 0.8721 | 0.1190 | 1.3575 | 0.0404 | 0.4602 | 0.0450 | 0.5130 |
| Constipation | 0.0032 | 0.0009 | 0.2675 | 0.0023 | 0.7034 | 0.0032 | 1.0083 | 0.0024 | 0.7500 | 0.0000 | 0.0000 | 0.0078 | 2.4477 |
| COPD | 0.0440 | 0.0176 | 0.3998 | 0.0424 | 0.9627 | 0.0199 | 0.4531 | 0.1327 | 3.0167 | 0.0005 | 0.0124 | 0.0166 | 0.3782 |
| Dementia | 0.0005 | 0.0003 | 0.5802 | 0.0004 | 0.7608 | 0.0004 | 0.8679 | 0.0009 | 1.7059 | 0.0003 | 0.5192 | 0.0006 | 1.2319 |
| Depression | 0.1095 | 0.0682 | 0.6225 | 0.0897 | 0.8193 | 0.0725 | 0.6619 | 0.1093 | 0.9983 | 0.0288 | 0.2633 | 0.2107 | 1.9243 |
| Diabetes | 0.1035 | 0.0512 | 0.4951 | 0.2170 | 2.0964 | 0.0927 | 0.8959 | 0.0938 | 0.9064 | 0.0501 | 0.4839 | 0.0254 | 0.2451 |
| Drug abuse | 0.0001 | 0.0001 | 1.2773 | 0.0000 | 0.0000 | 0.0000 | 0.0000 | 0.0000 | 0.0000 | 0.0000 | 0.0000 | 0.0006 | 5.9842 |
| Dyspepsia | 0.2015 | 0.1166 | 0.5786 | 0.1821 | 0.9036 | 0.1319 | 0.6546 | 0.2112 | 1.0480 | 0.0906 | 0.4496 | 0.3475 | 1.7245 |
| Endometriosis | 0.0199 | 0.0129 | 0.6473 | 0.0141 | 0.7076 | 0.0115 | 0.5801 | 0.0147 | 0.7412 | 0.0080 | 0.4007 | 0.0429 | 2.1552 |
| Epilepsy | 0.0124 | 0.0098 | 0.7897 | 0.0093 | 0.7529 | 0.0091 | 0.7374 | 0.0257 | 2.0705 | 0.0007 | 0.0585 | 0.0118 | 0.9520 |
| Glaucoma | 0.0291 | 0.0236 | 0.8122 | 0.0329 | 1.1289 | 0.0232 | 0.7989 | 0.0370 | 1.2711 | 0.0128 | 0.4395 | 0.0320 | 1.0997 |
| Heart failure | 0.0030 | 0.0024 | 0.8014 | 0.0035 | 1.1736 | 0.0023 | 0.7688 | 0.0056 | 1.8503 | 0.0006 | 0.2125 | 0.0008 | 0.2653 |
| Hepatitis | 0.0038 | 0.0036 | 0.9380 | 0.0022 | 0.5787 | 0.0037 | 0.9735 | 0.0019 | 0.4882 | 0.0012 | 0.3286 | 0.0100 | 2.6230 |
| Hypertension | 0.5476 | 0.4390 | 0.8017 | 1.0000 | 1.8262 | 0.4979 | 0.9092 | 0.1546 | 0.2823 | 1.0000 | 1.8262 | 0.2637 | 0.4815 |
| IBD | 0.0161 | 0.0117 | 0.7271 | 0.0147 | 0.9108 | 0.0050 | 0.3083 | 0.0354 | 2.1982 | 0.0046 | 0.2868 | 0.0141 | 0.8778 |
| IBS | 0.0614 | 0.0250 | 0.4073 | 0.0368 | 0.5995 | 0.0259 | 0.4214 | 0.0373 | 0.6073 | 0.0119 | 0.1940 | 0.1711 | 2.7869 |
| Intestinal disease | 0.0431 | 0.0231 | 0.5353 | 0.0375 | 0.8705 | 0.0204 | 0.4728 | 0.0428 | 0.9930 | 0.0218 | 0.5066 | 0.0822 | 1.9073 |
| Kidney disease | 0.0055 | 0.0033 | 0.5910 | 0.0116 | 2.1172 | 0.0022 | 0.4039 | 0.0044 | 0.8005 | 0.0004 | 0.0816 | 0.0035 | 0.6382 |
| Liver disease | 0.0045 | 0.0034 | 0.7633 | 0.0032 | 0.7080 | 0.0038 | 0.8403 | 0.0104 | 2.3033 | 0.0019 | 0.4290 | 0.0036 | 0.8012 |
| Meniere’s disease | 0.0076 | 0.0032 | 0.4249 | 0.0078 | 1.0258 | 0.0053 | 0.7019 | 0.0097 | 1.2785 | 0.0060 | 0.7849 | 0.0109 | 1.4312 |
| Migraine | 0.0611 | 0.0438 | 0.7176 | 0.0318 | 0.5210 | 0.0287 | 0.4693 | 0.0387 | 0.6327 | 0.0114 | 0.1865 | 0.1578 | 2.5821 |
| Multiple sclerosis | 0.0063 | 0.0045 | 0.7131 | 0.0066 | 1.0521 | 0.0039 | 0.6241 | 0.0095 | 1.5047 | 0.0001 | 0.0122 | 0.0068 | 1.0814 |
| Osteoporosis | 0.0821 | 0.0837 | 1.0196 | 0.0538 | 0.6555 | 0.0490 | 0.5967 | 0.1734 | 2.1126 | 0.0225 | 0.2745 | 0.0818 | 0.9968 |
| Pain | 0.4061 | 0.3033 | 0.7468 | 0.1857 | 0.4573 | 0.2975 | 0.7325 | 0.4170 | 1.0269 | 1.0000 | 2.4624 | 0.6057 | 1.4914 |
| Parkinson’s disease | 0.0035 | 0.0029 | 0.8425 | 0.0029 | 0.8350 | 0.0009 | 0.2610 | 0.0089 | 2.5337 | 0.0000 | 0.0000 | 0.0030 | 0.8579 |
| Polycystic ovary | 0.0011 | 0.0011 | 0.9920 | 0.0005 | 0.4716 | 0.0020 | 1.7919 | 0.0000 | 0.0000 | 0.0006 | 0.5885 | 0.0026 | 2.3611 |
| Psoriasis | 0.0548 | 0.0248 | 0.4517 | 0.0384 | 0.7004 | 0.0261 | 0.4767 | 0.1059 | 1.9328 | 0.0131 | 0.2384 | 0.0785 | 1.4319 |
| PVD | 0.0050 | 0.0021 | 0.4167 | 0.0060 | 1.1934 | 0.0024 | 0.4720 | 0.0093 | 1.8628 | 0.0003 | 0.0665 | 0.0045 | 0.8940 |
| Schizophrenia | 0.0055 | 0.0016 | 0.2912 | 0.0047 | 0.8491 | 0.0093 | 1.6982 | 0.0089 | 1.6257 | 0.0000 | 0.0000 | 0.0051 | 0.9274 |
| Sinusitis | 0.0133 | 0.0090 | 0.6752 | 0.0074 | 0.5555 | 0.0035 | 0.2625 | 0.0203 | 1.5247 | 0.0009 | 0.0691 | 0.0260 | 1.9518 |
| Stroke | 0.0422 | 0.0234 | 0.5553 | 0.0753 | 1.7849 | 0.0289 | 0.6853 | 0.0489 | 1.1591 | 0.0143 | 0.3384 | 0.0218 | 0.5167 |
| Thyroid conditions | 0.2048 | 0.1020 | 0.4981 | 0.0793 | 0.3871 | 1.0000 | 4.8828 | 0.1118 | 0.5459 | 0.0000 | 0.0000 | 0.1132 | 0.5529 |

Abbreviations: CFS, Chronic Fatigue Syndrome; CHD, Coronary Heart Disease; COPD, Chronic Obstructive Pulmonary Disease; IBD, Inflammatory Bowel Disease; IBS, Irritable Bowel Syndrome; O/E, Observed/Expected; P, Probability; PVD, Peripheral Vascular Disease

# Table S10. Probabilities and Observed vs Expected Ratios within 6 clusters derived in the training sample in men aged 40-59

| **Condition** |  | **Hypertension, pain & CHD** | | **Asthma & COPD** | | **Depression, pain, dyspepsia** | | **Psoriasis, asthma & arthritis** | | **Diabetes, hypertension & CHD** | | **Pain, dyspepsia & cancer** | |
| --- | --- | --- | --- | --- | --- | --- | --- | --- | --- | --- | --- | --- | --- |
|  | **Expected** | **P** | **O/E** | **P** | **O/E** | **P** | **O/E** | **P** | **O/E** | **P** | **O/E** | **P** | **O/E** |
| Alcohol problems | 0.0120 | 0.0091 | 0.7562 | 0.0061 | 0.5077 | 0.0495 | 4.1289 | 0.0043 | 0.3603 | 0.0033 | 0.276 | 0.0158 | 1.3167 |
| Anaemia | 0.0035 | 0.0023 | 0.6678 | 0.0013 | 0.3838 | 0.0038 | 1.0958 | 0.0038 | 1.0752 | 0.0027 | 0.7677 | 0.0079 | 2.2591 |
| Anorexia | 0.0005 | 0.0002 | 0.4035 | 0.0002 | 0.4253 | 0.0027 | 5.3882 | 0.0003 | 0.5184 | 0.0000 | 0.0000 | 0.0011 | 2.1892 |
| Anxiety | 0.0509 | 0.0360 | 0.7078 | 0.0262 | 0.5152 | 0.1870 | 3.6742 | 0.0270 | 0.5305 | 0.0173 | 0.3397 | 0.0745 | 1.4630 |
| Arthritis | 0.0337 | 0.0314 | 0.9311 | 0.0221 | 0.6555 | 0.0159 | 0.4711 | 0.0645 | 1.9148 | 0.0211 | 0.627 | 0.0553 | 1.6417 |
| Asthma | 0.2807 | 0.0006 | 0.0022 | 1.0000 | 3.5625 | 0.1404 | 0.5003 | 0.4547 | 1.6199 | 0.0968 | 0.345 | 0.0287 | 0.1021 |
| Atrial fibrillation | 0.0161 | 0.0199 | 1.2384 | 0.0122 | 0.7562 | 0.0043 | 0.2648 | 0.0085 | 0.5305 | 0.0099 | 0.6139 | 0.0339 | 2.1063 |
| Bronchiectasis | 0.0031 | 0.0016 | 0.5063 | 0.0088 | 2.8378 | 0.0005 | 0.1762 | 0.0023 | 0.7514 | 0.0000 | 0.0000 | 0.0050 | 1.6248 |
| Cancer | 0.0884 | 0.1054 | 1.1925 | 0.0661 | 0.7477 | 0.0558 | 0.6310 | 0.0482 | 0.5457 | 0.0471 | 0.5329 | 0.1668 | 1.8874 |
| CFS | 0.0088 | 0.0061 | 0.6891 | 0.0054 | 0.6146 | 0.0267 | 3.0299 | 0.0050 | 0.5701 | 0.0027 | 0.3062 | 0.0170 | 1.9339 |
| CHD | 0.1171 | 0.1675 | 1.4301 | 0.0711 | 0.6073 | 0.0577 | 0.4926 | 0.0242 | 0.2066 | 0.1755 | 1.4987 | 0.1147 | 0.9791 |
| Constipation | 0.0010 | 0.0005 | 0.4968 | 0.0005 | 0.5280 | 0.0010 | 1.0283 | 0.0005 | 0.4916 | 0.0000 | 0.0000 | 0.0028 | 2.8359 |
| COPD | 0.0388 | 0.0256 | 0.6595 | 0.0707 | 1.8229 | 0.0344 | 0.8875 | 0.0235 | 0.6066 | 0.0195 | 0.5015 | 0.0579 | 1.4919 |
| Dementia | 0.0003 | 0.0000 | 0.0000 | 0.0004 | 1.3316 | 0.0018 | 6.0935 | 0.0000 | 0.0000 | 0.0000 | 0.0000 | 0.0005 | 1.8063 |
| Depression | 0.1468 | 0.0706 | 0.4809 | 0.0761 | 0.5184 | 1.0000 | 6.8120 | 0.0498 | 0.3391 | 0.0738 | 0.5027 | 0.0016 | 0.0109 |
| Diabetes | 0.1670 | 0.0098 | 0.0588 | 0.0538 | 0.3220 | 0.0454 | 0.2719 | 0.0178 | 0.1067 | 1.0000 | 5.9880 | 0.0353 | 0.2114 |
| Drug abuse | 0.0015 | 0.0006 | 0.3776 | 0.0006 | 0.3741 | 0.0063 | 4.1987 | 0.0000 | 0.0000 | 0.0003 | 0.2012 | 0.0041 | 2.7455 |
| Dyspepsia | 0.1914 | 0.1827 | 0.9544 | 0.1688 | 0.8822 | 0.2031 | 1.0613 | 0.1042 | 0.5443 | 0.0907 | 0.4736 | 0.3634 | 1.8986 |
| Epilepsy | 0.0248 | 0.0222 | 0.8942 | 0.0203 | 0.8171 | 0.0297 | 1.1956 | 0.0135 | 0.5448 | 0.0086 | 0.3467 | 0.0499 | 2.0119 |
| Glaucoma | 0.0171 | 0.0176 | 1.0295 | 0.0139 | 0.8151 | 0.0140 | 0.8165 | 0.0085 | 0.4968 | 0.0164 | 0.9589 | 0.0286 | 1.6725 |
| Heart failure | 0.0063 | 0.0076 | 1.2052 | 0.0039 | 0.6141 | 0.0027 | 0.4286 | 0.0032 | 0.5126 | 0.0074 | 1.1704 | 0.0123 | 1.9521 |
| Hepatitis | 0.0092 | 0.0053 | 0.5717 | 0.0074 | 0.7992 | 0.0182 | 1.9814 | 0.0044 | 0.4734 | 0.0021 | 0.2299 | 0.0230 | 2.4953 |
| Hypertension | 0.5736 | 1.0000 | 1.7434 | 0.4580 | 0.7985 | 0.2749 | 0.4793 | 0.2294 | 0.3999 | 0.8328 | 1.4519 | 0.0000 | 0.0000 |
| IBD | 0.0195 | 0.0173 | 0.8872 | 0.0184 | 0.9447 | 0.0118 | 0.6052 | 0.0188 | 0.9639 | 0.0077 | 0.3963 | 0.0377 | 1.9331 |
| IBS | 0.0398 | 0.0321 | 0.8077 | 0.0280 | 0.7047 | 0.0590 | 1.4831 | 0.0409 | 1.0279 | 0.0079 | 0.1989 | 0.0853 | 2.1433 |
| Intestinal disease | 0.0172 | 0.0181 | 1.0549 | 0.0120 | 0.6983 | 0.0166 | 0.9641 | 0.0105 | 0.6120 | 0.0101 | 0.5862 | 0.0360 | 2.0912 |
| Kidney disease | 0.0105 | 0.0194 | 1.8470 | 0.0058 | 0.5527 | 0.0008 | 0.0743 | 0.0000 | 0.0000 | 0.0159 | 1.5148 | 0.0074 | 0.7004 |
| Liver disease | 0.0058 | 0.0058 | 1.0065 | 0.0027 | 0.4676 | 0.0032 | 0.5442 | 0.0053 | 0.9074 | 0.0068 | 1.1765 | 0.0119 | 2.0580 |
| Meniere’s disease | 0.0042 | 0.0049 | 1.1753 | 0.0029 | 0.6968 | 0.0038 | 0.9111 | 0.0018 | 0.4247 | 0.0018 | 0.4190 | 0.0089 | 2.1301 |
| Migraine | 0.0409 | 0.0303 | 0.7403 | 0.0269 | 0.6574 | 0.0479 | 1.1714 | 0.0334 | 0.8164 | 0.0065 | 0.1598 | 0.1106 | 2.7036 |
| Multiple sclerosis | 0.0061 | 0.0053 | 0.8671 | 0.0061 | 0.9930 | 0.0053 | 0.8656 | 0.0039 | 0.6376 | 0.0038 | 0.6285 | 0.0110 | 1.7971 |
| Osteoporosis | 0.0098 | 0.0067 | 0.6843 | 0.0096 | 0.9785 | 0.0081 | 0.8305 | 0.0061 | 0.6255 | 0.0047 | 0.4839 | 0.0251 | 2.5599 |
| Pain | 0.3286 | 0.3688 | 1.1224 | 0.2561 | 0.7792 | 0.3331 | 1.0138 | 0.2258 | 0.6872 | 0.1827 | 0.5558 | 0.5261 | 1.6011 |
| Parkinson’s disease | 0.0023 | 0.0020 | 0.8736 | 0.0020 | 0.8589 | 0.0011 | 0.4584 | 0.0010 | 0.4224 | 0.0010 | 0.4357 | 0.0067 | 2.9045 |
| Prostate disorders | 0.0353 | 0.0356 | 1.0079 | 0.0242 | 0.6861 | 0.0246 | 0.6969 | 0.0256 | 0.7264 | 0.0135 | 0.3819 | 0.0791 | 2.2415 |
| Psoriasis | 0.1076 | 0.0336 | 0.3118 | 0.0092 | 0.0852 | 0.0553 | 0.5137 | 1.0000 | 9.2937 | 0.0335 | 0.3116 | 0.0072 | 0.0671 |
| PVD | 0.0053 | 0.0054 | 1.0261 | 0.0023 | 0.4351 | 0.0042 | 0.8006 | 0.0026 | 0.4904 | 0.0040 | 0.7603 | 0.0120 | 2.2629 |
| Schizophrenia | 0.0151 | 0.0115 | 0.7641 | 0.0107 | 0.7112 | 0.0285 | 1.8898 | 0.0045 | 0.2970 | 0.0126 | 0.8323 | 0.0291 | 1.9257 |
| Sinusitis | 0.0153 | 0.0092 | 0.6037 | 0.0158 | 1.0350 | 0.0150 | 0.9781 | 0.0120 | 0.7817 | 0.0029 | 0.1913 | 0.0359 | 2.3441 |
| Stroke | 0.0430 | 0.0627 | 1.4592 | 0.0233 | 0.5426 | 0.0290 | 0.6735 | 0.0114 | 0.2651 | 0.0477 | 1.1098 | 0.0524 | 1.2188 |
| Thyroid conditions | 0.0436 | 0.0377 | 0.8639 | 0.0323 | 0.7411 | 0.0336 | 0.7697 | 0.0256 | 0.5882 | 0.0470 | 1.0777 | 0.0793 | 1.8191 |

Abbreviations: CFS, Chronic Fatigue Syndrome; CHD, Coronary Heart Disease; COPD, Chronic Obstructive Pulmonary Disease; IBD, Inflammatory Bowel Disease; IBS, Irritable Bowel Syndrome; O/E, Observed/Expected; P, Probability; PVD, Peripheral Vascular Disease

# Table S11. Probabilities and Observed vs Expected Ratios within 5 clusters derived in the training sample in men aged 60-70

| **Condition** |  | **Pain, dyspepsia & CHD** | | **Asthma, COPD & psoriasis** | | **Hypertension, pain & CHD** | | **Diabetes, hypertension & CHD** | | **Cancer** | |
| --- | --- | --- | --- | --- | --- | --- | --- | --- | --- | --- | --- |
|  | **Expected** | **P** | **O/E** | **P** | **O/E** | **P** | **O/E** | **P** | **O/E** | **P** | **O/E** |
| Alcohol problems | 0.0038 | 0.0078 | 2.0422 | 0.0033 | 0.8795 | 0.0038 | 0.9894 | 0.0005 | 0.1423 | 0.0031 | 0.8195 |
| Anaemia | 0.0049 | 0.0093 | 1.8892 | 0.0040 | 0.8223 | 0.0041 | 0.8274 | 0.0052 | 1.0617 | 0.0037 | 0.7498 |
| Anorexia | 0.0001 | 0.0003 | 2.8949 | 0.0000 | 0.0000 | 0.0001 | 0.7002 | 0.0000 | 0.0000 | 0.0000 | 0.0000 |
| Anxiety | 0.0262 | 0.0541 | 2.0663 | 0.0160 | 0.6107 | 0.0239 | 0.9140 | 0.0089 | 0.3412 | 0.0189 | 0.7219 |
| Arthritis | 0.0332 | 0.0513 | 1.5466 | 0.0312 | 0.9390 | 0.0315 | 0.9473 | 0.0153 | 0.4621 | 0.0279 | 0.8389 |
| Asthma | 0.1751 | 0.0417 | 0.2380 | 1.0000 | 5.7110 | 0.0502 | 0.2867 | 0.0845 | 0.4824 | 0.0001 | 0.0008 |
| Atrial fibrillation | 0.0337 | 0.0523 | 1.5527 | 0.0195 | 0.5772 | 0.0368 | 1.0933 | 0.0204 | 0.6055 | 0.0265 | 0.7875 |
| Bronchiectasis | 0.0049 | 0.0085 | 1.7412 | 0.0145 | 2.9521 | 0.0023 | 0.4706 | 0.0008 | 0.1548 | 0.0039 | 0.7945 |
| Cancer | 0.1561 | 0.0365 | 0.2339 | 0.1499 | 0.9602 | 0.0000 | 0.0000 | 0.0716 | 0.4586 | 1.0000 | 6.4061 |
| CFS | 0.0034 | 0.0074 | 2.1626 | 0.0026 | 0.7524 | 0.0026 | 0.7678 | 0.0012 | 0.3488 | 0.0021 | 0.6088 |
| CHD | 0.2237 | 0.2366 | 1.0575 | 0.1387 | 0.6202 | 0.2485 | 1.1108 | 0.2833 | 1.2665 | 0.1378 | 0.6160 |
| Constipation | 0.0011 | 0.0017 | 1.5498 | 0.0010 | 0.8871 | 0.0007 | 0.6670 | 0.0010 | 0.8906 | 0.0017 | 1.5320 |
| COPD | 0.0572 | 0.0718 | 1.2559 | 0.1317 | 2.3018 | 0.0412 | 0.7206 | 0.0277 | 0.4845 | 0.0373 | 0.6525 |
| Dementia | 0.0010 | 0.0034 | 3.4012 | 0.0001 | 0.0659 | 0.0008 | 0.8154 | 0.0000 | 0.0000 | 0.0004 | 0.3651 |
| Depression | 0.0698 | 0.1291 | 1.8503 | 0.0544 | 0.7791 | 0.0656 | 0.9404 | 0.0375 | 0.5369 | 0.0418 | 0.5983 |
| Diabetes | 0.1974 | 0.0537 | 0.2718 | 0.0728 | 0.3690 | 0.0629 | 0.3188 | 1.0000 | 5.0659 | 0.0562 | 0.2847 |
| Drug abuse | 0.0003 | 0.0005 | 1.6025 | 0.0005 | 1.8332 | 0.0003 | 0.9931 | 0.0000 | 0.0000 | 0.0002 | 0.6462 |
| Dyspepsia | 0.1845 | 0.3185 | 1.7263 | 0.1528 | 0.8281 | 0.1795 | 0.9727 | 0.0814 | 0.4414 | 0.1381 | 0.7485 |
| Epilepsy | 0.0140 | 0.0267 | 1.9084 | 0.0080 | 0.5689 | 0.0139 | 0.9939 | 0.0054 | 0.3842 | 0.0132 | 0.9439 |
| Glaucoma | 0.0347 | 0.0541 | 1.5598 | 0.0232 | 0.6677 | 0.0327 | 0.9413 | 0.0250 | 0.7217 | 0.0307 | 0.8852 |
| Heart failure | 0.0059 | 0.0093 | 1.5681 | 0.0039 | 0.6569 | 0.0063 | 1.0629 | 0.0050 | 0.8543 | 0.0025 | 0.4254 |
| Hepatitis | 0.0041 | 0.0102 | 2.4780 | 0.0025 | 0.6020 | 0.0028 | 0.6827 | 0.0012 | 0.3003 | 0.0035 | 0.8560 |
| Hypertension | 0.6589 | 0.0026 | 0.0040 | 0.4709 | 0.7146 | 1.0000 | 1.5177 | 0.8066 | 1.2242 | 0.6021 | 0.9138 |
| IBD | 0.0157 | 0.0287 | 1.8306 | 0.0151 | 0.9592 | 0.0124 | 0.7911 | 0.0096 | 0.6126 | 0.0125 | 0.7938 |
| IBS | 0.0223 | 0.0500 | 2.2442 | 0.0194 | 0.8710 | 0.0201 | 0.9017 | 0.0028 | 0.1271 | 0.0125 | 0.5596 |
| Intestinal disease | 0.0234 | 0.0405 | 1.7288 | 0.0203 | 0.8686 | 0.0235 | 1.0031 | 0.0101 | 0.4316 | 0.0156 | 0.6668 |
| Kidney disease | 0.0076 | 0.0045 | 0.5933 | 0.0034 | 0.4412 | 0.0108 | 1.4195 | 0.0093 | 1.2285 | 0.0087 | 1.1453 |
| Liver disease | 0.0033 | 0.0051 | 1.5530 | 0.0009 | 0.2604 | 0.0024 | 0.7210 | 0.0040 | 1.2130 | 0.0026 | 0.8004 |
| Meniere’s disease | 0.0056 | 0.0110 | 1.9709 | 0.0044 | 0.7873 | 0.0055 | 0.9890 | 0.0013 | 0.2360 | 0.0041 | 0.7235 |
| Migraine | 0.0215 | 0.0455 | 2.1150 | 0.0162 | 0.7547 | 0.0198 | 0.9216 | 0.0016 | 0.0732 | 0.0145 | 0.6742 |
| Multiple sclerosis | 0.0025 | 0.0062 | 2.4675 | 0.0011 | 0.4222 | 0.0024 | 0.9613 | 0.0015 | 0.6111 | 0.0013 | 0.5012 |
| Osteoporosis | 0.0113 | 0.0244 | 2.1560 | 0.0129 | 1.1456 | 0.0079 | 0.7012 | 0.0021 | 0.1900 | 0.0116 | 1.0287 |
| Pain | 0.3494 | 0.5090 | 1.4568 | 0.2605 | 0.7456 | 0.3788 | 1.0842 | 0.2087 | 0.5973 | 0.2646 | 0.7574 |
| Parkinson’s disease | 0.0065 | 0.0126 | 1.9401 | 0.0045 | 0.6867 | 0.0057 | 0.8697 | 0.0020 | 0.3096 | 0.0071 | 1.0854 |
| Prostate disorders | 0.1164 | 0.2229 | 1.9149 | 0.0860 | 0.7388 | 0.1159 | 0.9955 | 0.0421 | 0.3615 | 0.0765 | 0.6573 |
| Psoriasis | 0.0636 | 0.0998 | 1.5685 | 0.0958 | 1.5055 | 0.0549 | 0.8629 | 0.0265 | 0.4171 | 0.0372 | 0.5844 |
| PVD | 0.0077 | 0.0104 | 1.3548 | 0.0039 | 0.5091 | 0.0081 | 1.0557 | 0.0074 | 0.9574 | 0.0049 | 0.6424 |
| Schizophrenia | 0.0062 | 0.0142 | 2.2865 | 0.0034 | 0.5479 | 0.0052 | 0.8327 | 0.0033 | 0.5395 | 0.0026 | 0.4272 |
| Sinusitis | 0.0105 | 0.0196 | 1.8710 | 0.0104 | 0.9894 | 0.0106 | 1.0098 | 0.0017 | 0.1653 | 0.0038 | 0.3593 |
| Stroke | 0.0701 | 0.0759 | 1.0828 | 0.0360 | 0.5141 | 0.0883 | 1.2594 | 0.0658 | 0.9386 | 0.0528 | 0.7525 |
| Thyroid conditions | 0.0483 | 0.0774 | 1.6021 | 0.0294 | 0.6085 | 0.0465 | 0.9632 | 0.0438 | 0.9071 | 0.0372 | 0.7706 |

Abbreviations: CFS, Chronic Fatigue Syndrome; CHD, Coronary Heart Disease; COPD, Chronic Obstructive Pulmonary Disease; IBD, Inflammatory Bowel Disease; IBS, Irritable Bowel Syndrome; O/E, Observed/Expected; P, Probability; PVD, Peripheral Vascular Disease

# Table S12. Association between multimorbidity clusters derived in the training sample with cancer, vascular and other-cause mortality in women in UK Biobank

|  | **Cancer mortality** | | **Vascular mortality** | | **Other-cause mortality** | |
| --- | --- | --- | --- | --- | --- | --- |
| **Disease clusters** | **Model A^a^**  **HR (95% CI)** | **Model B^b^**  **HR (95% CI)** | **Model A^a^**  **HR (95% CI)** | **Model B^b^**  **HR (95% CI)** | **Model A^a^**  **HR (95% CI)** | **Model B^b^**  **HR (95% CI)** |
| ***40-59 years old at baseline*** |  |  |  |  |  |  |
| No multimorbidity | 1.00 (Reference) | 1.00 (Reference) | 1.00 (Reference) | 1.00 (Reference) | 1.00 (Reference) | 1.00 (Reference) |
| Multimorbidity | 1.58 (1.46-1.70) | 1.50 (1.39-1.62) | 2.93 (2.49-3.44) | 2.53 (2.14-2.99) | 2.84 (2.53-3.18) | 2.49 (2.22-2.80) |
| Hypertension (100%), Diabetes (18%), CHD (7%) | 1.53 (1.37-1.71) | 1.45 (1.29-1.62) | 3.89 (3.18-4.76) | 3.32 (2.69-4.11) | 3.51 (3.03-4.06) | 3.06 (2.62-3.57) |
| Asthma (100%), Psoriasis (17%), COPD (7%) | 1.20 (1.01-1.43) | 1.14 (0.96-1.36) | 2.80 (2.06-3.79) | 2.37 (1.74-3.22) | 2.87 (2.33-3.53) | 2.53 (2.05-3.12) |
| Depression (38%), Cancer (27%), Dyspepsia (23%) | 2.51 (2.18-2.88) | 2.34 (2.03-2.69) | 2.98 (2.13-4.16) | 2.54 (1.81-3.57) | 2.76 (2.17-3.51) | 2.42 (1.90-3.08) |
| Pain (100%), Depression (25%), Dyspepsia (23%) | 1.54 (1.29-1.83) | 1.43 (1.20-1.70) | 1.98 (1.34-2.93) | 1.63 (1.10-2.42) | 2.72 (2.14-3.46) | 2.34 (1.84-2.99) |
| Thyroid conditions (100%), Pain (22%), Hypertension (21%) | 1.47 (1.22-1.77) | 1.41 (1.17-1.70) | 2.11 (1.41-3.15) | 1.88 (1.25-2.81) | 2.11 (1.59-2.79) | 1.94 (1.46-2.58) |
| Migraine (100%), Pain (30%), Hypertension (20%) | 1.38 (1.11-1.72) | 1.36 (1.09-1.69) | 1.12 (0.60-2.11) | 1.08 (0.58-2.04) | 1.47 (1.01-2.15) | 1.41 (0.96-2.06) |
| ***60-70 years old at baseline*** |  |  |  |  |  |  |
| No multimorbidity | 1.00 (Reference) | 1.00 (Reference) | 1.00 (Reference) | 1.00 (Reference) | 1.00 (Reference) | 1.00 (Reference) |
| Multimorbidity | 1.34 (1.27-1.41) | 1.29 (1.22-1.36) | 2.11 (1.92-2.31) | 1.92 (1.75-2.11) | 2.09 (1.95-2.23) | 2.02 (1.89-2.16) |
| Hypertension (100%), Asthma (23%), Diabetes (22%) | 1.27 (1.17-1.38) | 1.22 (1.12-1.33) | 3.15 (2.80-3.55) | 2.80 (2.47-3.16) | 2.53 (2.31-2.77) | 2.45 (2.23-2.69) |
| Pain (61%), Dyspepsia (35%), Depression (21%) | 0.94 (0.84-1.06) | 0.92 (0.82-1.03) | 1.36 (1.12-1.65) | 1.26 (1.04-1.53) | 1.47 (1.29-1.68) | 1.44 (1.26-1.65) |
| Cancer (100%), Osteoporosis (8%) | 2.35 (2.17-2.55) | 2.29 (2.12-2.48) | 1.41 (1.17-1.71) | 1.34 (1.10-1.62) | 1.61 (1.41-1.83) | 1.58 (1.39-1.81) |
| Thyroid conditions (100%) | 1.11 (0.99-1.24) | 1.06 (0.95-1.19) | 1.63 (1.35-1.96) | 1.46 (1.21-1.76) | 1.89 (1.67-2.15) | 1.84 (1.61-2.09) |
| Asthma (43%), Pain (42%), Dyspepsia (21%) | 1.27(1.14-1.42) | 1.20 (1.08-1.35) | 2.59 (2.21-3.04) | 2.34 (1.99-2.75) | 3.26 (2.93-3.62) | 3.06 (2.75-3.40) |
| Hypertension (100%), Pain (100%) | 0.89 (0.76-1.04) | 0.86 (0.73-1.00) | 1.67 (1.33-2.09) | 1.51 (1.20-1.90) | 1.44 (1.21-1.71) | 1.46 (1.22-1.74) |

Abbreviations: CHD, Coronary Heart Disease; CI, Confidence Interval; COPD, Chronic Obstructive Pulmonary Disease; HR, Hazard Ratio

^a^ Adjusted for age, sex, ethnicity, Townsend deprivation index and education

^b^ Adjusted for age, sex, ethnicity, Townsend deprivation index, education, body mass index, smoking, alcohol intake and physical activity

**Table S13. Association between multimorbidity clusters derived in the training sample with cancer, vascular and other-cause mortality in men in UK Biobank**

|  | **Cancer mortality** | | **Vascular mortality** | | **Other-cause mortality** | |
| --- | --- | --- | --- | --- | --- | --- |
| **Disease clusters** | **Model A^a^**  **HR (95% CI)** | **Model B^b^**  **HR (95% CI)** | **Model A^a^**  **HR (95% CI)** | **Model B^b^**  **HR (95% CI)** | **Model A^a^**  **HR (95% CI)** | **Model B^b^**  **HR (95% CI)** |
| ***40-59 years old at baseline*** |  |  |  |  |  |  |
| No multimorbidity | 1.00 (Reference) | 1.00 (Reference) | 1.00 (Reference) | 1.00 (Reference) | 1.00 (Reference) | 1.00 (Reference) |
| Multimorbidity | 1.56 (1.44-1.69) | 1.50 (1.38-1.62) | 2.81 (2.54-3.11) | 2.51 (2.26-2.79) | 2.71 (2.48-2.97) | 2.58 (2.35-2.83) |
| Hypertension (100%), Pain (37%), CHD (17%) | 1.49 (1.32-1.68) | 1.42 (1.26-1.61) | 2.76 (2.38-3.19) | 2.50 (2.16-2.91) | 2.19 (1.89-2.52) | 2.09 (1.81-2.42) |
| Asthma (100%), COPD (7%) | 1.45 (1.24-1.70) | 1.41 (1.21-1.66) | 1.96 (1.59-2.41) | 1.83 (1.48-2.26) | 2.58 (2.19-3.03) | 2.48 (2.11-2.93) |
| Diabetes (100%), Hypertension (83%), CHD (18%) | 1.82 (1.55-2.14) | 1.72 (1.46-2.03) | 5.87 (5.05-6.81) | 4.94 (4.21-5.79) | 4.37 (3.77-5.07) | 4.22 (3.60-4.94) |
| Pain (53%), Dyspepsia (36%), Cancer (17%) | 1.78 (1.51-2.10) | 1.72 (1.46-2.02) | 1.92 (1.51-2.44) | 1.78 (1.40-2.27) | 2.44 (2.01-2.96) | 2.28 (1.88-2.77) |
| Psoriasis (100%), Asthma (45%), Arthritis (6%) | 1.28 (0.98-1.68) | 1.26 (0.97-1.65) | 1.36 (0.91-2.02) | 1.33 (0.90-1.99) | 2.04 (1.53-2.73) | 2.01 (1.50-2.68) |
| Depression (100%), Pain (33%), Dyspepsia (20%) | 1.49 (1.16-1.92) | 1.35 (1.05-1.74) | 2.00 (1.45-2.76) | 1.72 (1.24-2.38) | 3.11 (2.47-3.93) | 2.79 (2.21-3.53) |
| ***60-70 years old at baseline*** |  |  |  |  |  |  |
| No multimorbidity | 1.00 (Reference) | 1.00 (Reference) | 1.00 (Reference) | 1.00 (Reference) | 1.00 (Reference) | 1.00 (Reference) |
| Multimorbidity | 1.32 (1.26-1.39) | 1.28 (1.22-1.34) | 2.13 (2.00-2.27) | 1.96 (1.84-2.09) | 2.09 (1.95-2.23) | 1.72 (1.63-1.82) |
| Hypertension (100%), Pain (38%), CHD (25%) | 1.04 (0.97-1.11) | 1.00 (0.93-1.07) | 2.06 (1.89-2.23) | 1.94 (1.78-2.11) | 1.53 (1.42-1.66) | 1.54 (1.43-1.66) |
| Pain (51%), Dyspepsia (32%), CHD (24%) | 1.03 (0.94-1.14) | 1.01 (0.91-1.11) | 1.58 (1.40-1.79) | 1.52 (1.35-1.72) | 1.74 (1.58-1.92) | 1.68 (1.53-1.85) |
| Diabetes (100%), Hypertension (81%), CHD (28%) | 1.52 (1.40-1.66) | 1.41 (1.29-1.54) | 3.42 (3.11-3.75) | 2.93 (2.66-3.24) | 2.53 (2.32-2.76 | 2.36 (2.15-2.58) |
| Asthma (100%), COPD (13%), Psoriasis (10%) | 1.24 (1.13-1.37) | 1.23 (1.12-1.35) | 1.82 (1.61-2.06) | 1.79 (1.58-2.02) | 1.74 (1.58-1.93) | 1.74 (1.57-1.93) |
| Cancer (100%) | 2.51 (2.32-2.71) | 2.44 (2.26-2.65) | 1.68 (1.46-1.93) | 1.62 (1.40-1.86) | 1.45 (1.28-1.64) | 1.45 (1.28-1.64) |

Abbreviations: CHD, Coronary Heart Disease; CI, Confidence Interval; COPD, Chronic Obstructive Pulmonary Disease; HR, Hazard Ratio

^a^ Adjusted for age, sex, ethnicity, Townsend deprivation index and education

^b^ Adjusted for age, sex, ethnicity, Townsend deprivation index, education, body mass index, smoking, alcohol intake and physical activity

**Table S14. Association between multimorbidity clusters derived in the test sample with all-cause mortality in women in**

**UK Biobank**

| **Disease clusters** | **N** | **Deaths** | **Median morbidities (IQR)** | **All-cause mortality** | |
| --- | --- | --- | --- | --- | --- |
|  |  |  |  | **Model A^a^**  **HR (95% CI)** | **Model B^b^**  **HR (95% CI)** |
| ***40-59 years old at baseline*** |  |  |  |  |  |
| No multimorbidity | 116,714 | 3,017 | 0 (0-1) | 1.00 (Reference) | 1.00 (Reference) |
| Multimorbidity | 8,378 | 506 | 2 (2-3) | 2.00 (1.82-2.20) | 1.86 (1.69-2.05) |
| Hypertension (100%), Pain (31%), Asthma (19%) | 2,825 | 200 | 2 (2-3) | 2.15 (1.86-2.49) | 1.98 (1.71-2.30) |
| Pain (55%), Migraine (24%), Dyspepsia (21%) | 1,654 | 66 | 2 (2-2) | 1.39 (1.09-1.77) | 1.32 (1.03-1.69) |
| Asthma (100%), Pain (29%), Psoriasis (16%) | 1,533 | 67 | 2 (2-3) | 1.62 (1.27-2.07) | 1.50 (1.18-1.91) |
| Cancer (100%), Asthma (26%), Hypertension (24%) | 1,125 | 119 | 2 (2-3) | 3.45 (2.87-4.15) | 3.20 (2.66-3.85) |
| Depression (100%), Pain (36%), Dyspepsia (17%) | 934 | 38 | 2 (2-3) | 1.47 (1.07-2.03) | 1.30 (1.08-1.09) |
| Thyroid conditions (47%), Arthritis (25%), Dyspepsia (21%) | 305 | 17 | 2 (2-3) | 1.92 (1.19-3.09) | 1.80 (1.11-2.90) |
| ***60-70 years old at baseline*** |  |  |  |  |  |
| No multimorbidity | 64,454 | 5,408 | 1 (0-1) | 1.00 (Reference) | 1.00 (Reference) |
| Multimorbidity | 9,936 | 1,530 | 2 (2-3) | 1.77 (1.67-1.87) | 1.69 (1.60-1.79) |
| Hypertension (100%), Pain (37%), Diabetes (20%) | 3,749 | 610 | 2 (2-3) | 1.81 (1.66-1.97) | 1.72 (1.58-1.88) |
| Pain (65%), Dyspepsia (38%), Hypertension (29%) | 1,768 | 217 | 2 (2-3) | 1.40 (1.22-1.60) | 1.34 (1.17-1.54) |
| Cancer (100%), Hypertension (45%). Pain (29%) | 1,622 | 328 | 2 (2-3) | 2.48 (2.22-2.77) | 2.42 (2.16-2.71) |
| Thyroid conditions (100%), Hypertension (40%), Pain (26%) | 1,406 | 175 | 2 (2-3) | 1.42 (1.23-1.66) | 1.38 (1.19-1.60) |
| Asthma (100%), Pain (34%), Dyspepsia (18%) | 993 | 127 | 2 (2-3) | 1.50 (1.26-1.79) | 1.42 (1.19-1.69) |
| Osteoporosis (34%), Pain (26%), Arthritis (23%) | 400 | 72 | 2 (2-3) | 2.02 (1.60-2.55) | 1.89 (1.50-2.39) |

Abbreviations: CI, Confidence Interval; HR, Hazard Ratio; IQR, Interquartile Range

^a^ Adjusted for age, sex, ethnicity, Townsend deprivation index and education

^b^ Adjusted for age, sex, ethnicity, Townsend deprivation index, education, body mass index, smoking, alcohol intake and physical activity

**Table S15. Association between multimorbidity clusters derived in the test sample with all-cause mortality in men in UK Biobank**

| **Disease clusters** | **N** | **Deaths** | **Median morbidities (IQR)** | **All-cause mortality** | |
| --- | --- | --- | --- | --- | --- |
|  |  |  |  | **Model A^a^**  **HR (95% CI)** | **Model B^b^**  **HR (95% CI)** |
| ***40-59 years old at baseline*** |  |  |  |  |  |
| No multimorbidity | 96,766 | 3,889 | 0 (0-1) | 1.00 (Reference) | 1.00 (Reference) |
| Multimorbidity | 5,899 | 645 | 2 (2-3) | 2.22 (2.04-2.42) | 2.08 (1.91-2.27) |
| Hypertension (100%), Pain (37%), Dyspepsia (19%) | 1,936 | 214 | 2 (2-3) | 2.07 (1.80-2.38) | 1.90 (1.65-2.18) |
| Asthma (100%), Hypertension (33%), Pain (23%) | 1,372 | 95 | 2 (2-3) | 1.63 (1.33-2.00) | 1.64 (1.34-2.01) |
| Pain (51%), Dyspepsia (33%), Depression (23%) | 1,157 | 94 | 2 (2-2) | 1.71 (1.39-2.10) | 1.60 (1.30-1.96) |
| Diabetes (100%). Hypertension 81%), CHD (19%) | 808 | 151 | 2 (2-3) | 3.49 (2.96-4.12) | 3.17 (2.68-3.75) |
| Cancer (100%), Hypertension (50%), Pain (18%) | 384 | 69 | 2 (2-3) | 3.63 (2.86-4.61) | 3.35 (2.64-4.25) |
| Anxiety (100%), Depression (51%), Hypertension (32%) | 244 | 23 | 2 (2-3) | 2.14 (2.42-3.23) | 2.00 (1.33-3.01) |
| ***60-70 years old at baseline*** |  |  |  |  |  |
| No multimorbidity | 58,171 | 8,374 | 1 (0-1) | 1.00 (Reference) | 1.00 (Reference) |
| Multimorbidity | 8,808 | 2,189 | 2 (2-3) | 1.68 (1.61-1.77) | 1.61 (1.54-1.69) |
| Hypertension (100%), Pain (38%), CHD (25%) | 3,450 | 794 | 2 (2-3) | 1.53 (1.43-1.65) | 1.47 (1.36-1.58) |
| Pain (49%), Dyspepsia (31%), CHD (23%) | 1,545 | 338 | 2 (2-3) | 1.45 (1.30-1.62) | 1.40 (1.26-1.57) |
| Diabetes (100%), Hypertension (81%), CHD (29%) | 1,540 | 512 | 2 (2-3) | 2.29 (2.09-2.51) | 2.13 (1.94-2.33) |
| Asthma (100%), Hypertension (43%), Pain (26%) | 1,270 | 266 | 2 (2-3) | 1.43 (1.27-1.62) | 1.41 (1.24-1.59) |
| Cancer (100%), Hypertension (63%), Pain (28%) | 1,002 | 278 | 2 (2-3) | 1.94 (1.72-2.19) | 1.92 (1.70-2.17) |

Abbreviations: CHD, Coronary Heart Disease; CI, Confidence Interval; HR, Hazard Ratio; IQR, Interquartile Range

^a^ Adjusted for age, sex, ethnicity, Townsend deprivation index and education

^b^ Adjusted for age, sex, ethnicity, Townsend deprivation index, education, body mass index, smoking, alcohol intake and physical activity
